# Treatment Efficacy for Non-Cardiovascular Chest Pain: A Systematic Review and Meta-Analysis

**DOI:** 10.1371/journal.pone.0104722

**Published:** 2014-08-11

**Authors:** Jakob M. Burgstaller, Boris F. Jenni, Johann Steurer, Ulrike Held, Maria M. Wertli

**Affiliations:** 1 Horten Center for Patient Oriented Research and Knowledge Transfer, Department of Internal Medicine, University of Zurich, Zurich, Switzerland; 2 Cantonal Hospital Winterthur, Winterthur, Switzerland; The James Cook University Hospital, United Kingdom

## Abstract

**Background:**

Non-cardiovascular chest pain (NCCP) leads to impaired quality of life and is associated with a high disease burden. Upon ruling out cardiovascular disease, only vague recommendations exist for further treatment.

**Objectives:**

To summarize treatment efficacy for patients presenting with NCCP.

**Methods:**

Systematic review and meta-analysis. In July 2013, Medline, Web of Knowledge, Embase, EBSCOhost, Cochrane Reviews and Trials, and Scopus were searched. Hand and bibliography searches were also conducted. Randomized controlled trials (RCTs) evaluating non-surgical treatments in patients with NCCP were included. Exclusion criteria were poor study quality and small sample size (<10 patients per group).

**Results:**

Thirty eligible RCT’s were included. Most studies assessed PPI efficacy for gastroesophageal reflux disorders (GERD, n = 10). Two RCTs included musculoskeletal chest pain, seven psychotropic drugs, and eleven various psychological interventions. Study quality was high in five RCTs and acceptable in 25. PPI treatment in patients with GERD (5 RCTs, 192 patients) was more effective than placebo [pooled OR 11.7 (95% CI 5.5 to 25.0, heterogeneity I^2^ = 6.1%)]. The pooled OR in GERD negative patients (4 RCTs, 156 patients) was 0.8 (95% CI 0.2 to 2.8, heterogeneity I^2^ = 50.4%). In musculoskeletal NCCP (2 RCTs, 229 patients) manual therapy was more effective than usual care but not than home exercise [pooled mean difference 0.5 (95% CI −0.3 to 1.3, heterogeneity I^2^ = 46.2%)]. The findings for cognitive behavioral treatment, serotonin reuptake inhibitors, tricyclic antidepressants were mixed. Most evidence was available for cognitive behavioral treatment interventions.

**Limitations:**

Only a small number of studies were available.

**Conclusions:**

Timely diagnostic evaluation and treatment of the disease underlying NCCP is important. For patients with suspected GERD, high-dose treatment with PPI is effective. Only limited evidence was available for most prevalent diseases manifesting with chest pain. In patients with idiopathic NCCP, treatments based on cognitive behavioral principles might be considered.

## Background

In the United States, 6 million patients present to emergency departments with chest pain each year, at an annual cost of $8 billion [1], [2]. Sixty to ninety percent of the patients that present to emergency departments with chest pain have no underlying cardiovascular disease [3]–[6]. The proportion of patients with cardiovascular disease is higher in specialized units (cardiology emergency departments, CCU, ICU) [7] and lower in the primary care setting [6], [8]–[10]. After serious illnesses have been ruled out, physicians often assume that patients with non-cardiovascular chest pain (NCCP) have an excellent prognosis [11], [12]. However, patients with NCCP have a high disease burden; many patients that seek care for NCCP complain of persisting symptoms in a 4-year follow-up [13]. Furthermore, patients with non-cardiac chest pain experience an impaired quality of life and greater number of medical visits compared with patients with cardiac pain [14].

In patients with chest pain, the diagnostic work-up focuses primarily on cardiovascular disease and is often performed by cardiologists. Upon ruling out cardiovascular disease, only vague recommendations exist for further treatment, delaying appropriate treatment and causing uncertainty for patients [15]. A recent systematic synthesis of diagnostic tests [16] showed that patients with gastroesophageal reflux disorder (GERD) can be identified by their response to proton pump inhibitor (PPI) treatment, and certain clinical findings can guide clinicians to the most appropriate treatments (e.g., pain increase with movement or decrease on medication were associated with musculoskeletal chest pain). However, limited data are available regarding the efficacy of treatments for patients with NCCP.

The present systematic review aimed to summarize the current evidence about the efficacy of different treatments based on randomized controlled trials (RCTs) for patients that seek care for NCCP.

## Methods

### Literature search and study selection

This search, conducted in July 2013, followed the PRISMA statement [17]. We searched six databases: Medline (OvidSP), including In-Process & Other Non-Indexed Citations, Daily and OLDMEDLINE; Web of Knowledge, including Biosis and Web of Science; Embase (OvidSP); EBSCOhost, including CINAHL and PsycINFO; Cochrane Reviews and Trials; and Scopus. We used the following search terms as medical subject headings (MeSH terms) and other subject headings: ‘thoracic pain’, ‘chest pain’, ‘non-cardiac chest pain’, ‘atypical chest pain’, ‘musculoskeletal chest pain’, ‘esophageal chest pain’, and ‘thoracic spine pain’. The findings were limited to studies published in the last 20 years. We applied no limits regarding study setting or language. **Table S1** depicts two detailed search strategies.

To ensure search completeness, one reviewer (BJ) conducted a thorough search of the bibliographies of all included studies. Potential eligible references were also included in the full text review.

### Eligibility criteria

Eligible studies were randomized controlled trials (RCTs) published in the last 20 years. Inclusion criteria were studies reporting on patients aged ≥18 years seeking care for NCCP. NCCP was defined as chest pain after cardiac or other vascular disease (e.g., cardiovascular disease, aortic dissection, pulmonary embolism) had been ruled out. Studies with less than 10 patients per group were excluded.

### Study selection, data extraction, and synthesis

Two reviewers (MW and BJ) independently screened 5372 references by title and abstract. Both reviewers independently reviewed the full text of 62 studies that met the eligibility criteria. Disagreements were discussed and resolved by consensus or third party arbitration (JS). Researchers with specific language proficiencies reviewed non-English language references. When the same study was included in several publications without change in treatment, outcome, or follow-up, the most recent publication was chosen and missing information was added from previous publications.

All information regarding the treatment and control groups, treatment duration, follow-up duration, and patient population was extracted and grouped according to the disease investigated.

### Quality assessment

Study quality was assessed using the Scottish Intercollegiate Guidelines Network (SIGN) methodology checklist for RCTs [18]. Overall bias risk and study quality were rated according to the SIGN recommendations. The ratings included high quality (++; the majority of criteria met; little or no risk of bias; results unlikely to be changed by further research.), acceptable quality (+; most criteria met; some flaws in the study with an associated risk of bias; conclusions may change in the light of further studies), and low quality (0; either most criteria not met, or significant flaws relating to key aspects of study design; conclusions likely to change in the light of further studies).

It was not possible to include all studies in the meta-analysis because data was missing for some outcomes. It may have happened that the studies originally considered several different outcome measures, but only reported the measures that provided significant results. Copas et al. [19] refer to this as outcome reporting bias, which is defined as outcome reporting driven by the significance and/or direction of the effect size. All studies that were not included in the meta-analyses were assessed for a potential outcome reporting bias using the 9-item outcome reporting in trails (ORBIT) tool [20]. Risk of bias was rated from low (outcome of interest was not measured) to high [trial report states that outcome was analyzed, but only reports that the result was not significant (typically stating p>0.05)].

### Outcome

The outcome of primary interest was chest pain, including chest pain frequency and intensity.

We also assessed psychological outcome measures. In particular, we aimed to assess the efficacy of treatment interventions on anxiety, depression, and panic disorders. All measures were extracted, and validity of the outcome measure used was assessed.

### Statistical analysis

Descriptive statistics were used to summarize findings across all groups of diagnostic studies. These included number of patients, mean patient age, and gender distribution.

In order to summarize findings across studies, different pain score scales were re-scaled to a 0- to 10-point scale where necessary. In addition, the frequency scores were homogenized to present results on a monthly basis. To present counted pain events in the treatment and control arms as odds ratios, we used number of events and number of patients in both groups, with a cut-off of >50% improvement. To present changes from baseline to follow-up in the treatment and control arms as mean differences, we used mean change, standard deviation of change, and number of patients in both groups. If the necessary information was not directly available from the original publication, we derived these quantities following instructions described in the Cochrane Handbook [21]. We assumed a random effects model to obtain a pooled estimate of the effect if more than one trial was available in a subgroup. A restricted maximum-likelihood estimator was used to quantify the amount of heterogeneity.

Risk of bias was assessed using a funnel plot. Funnel plot asymmetry was assessed with the regression test proposed by Egger [22].

Analyses were performed using R statistical software and the “metafor” package [23], [24].

## Results

### Study selection

**Figure 1:**
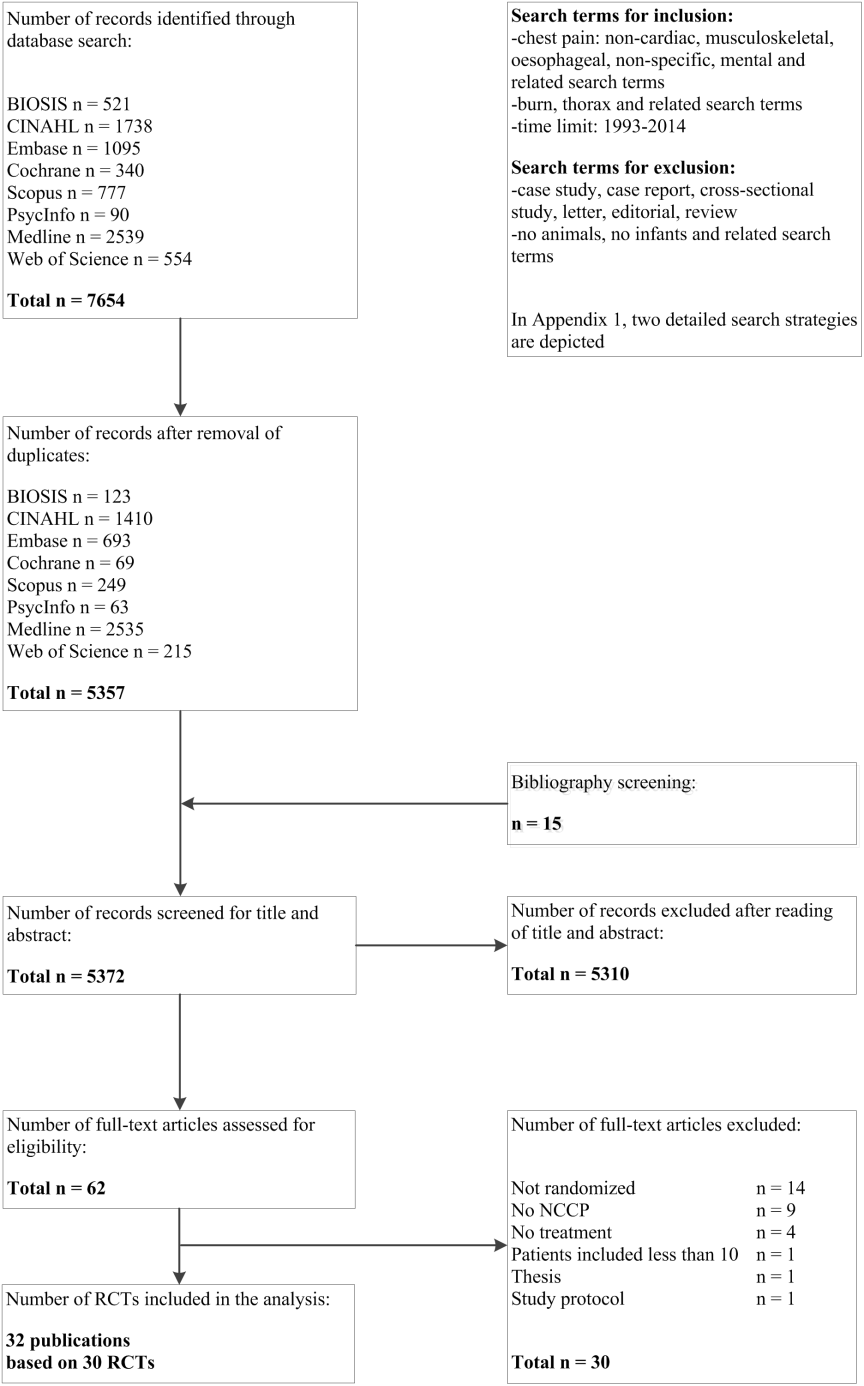
Study flow.

Figure 1 summarizes the search and inclusion process. Out of 5372 records, 62 were reviewed in full text, resulting in the exclusion of 5310 studies. In total, the analysis included 32 publications based on 30 RCTs. Reasons for the exclusion of 30 publications are provided in Figure 1.

### Study characteristics

Table 1 presents the study characteristics and included patients. Ten RCTs (33%) included patients with underlying gastrointestinal cause. Most RCTs (n = 8, 1037 patients) evaluated the treatment efficacy of a PPI in patients with NCCP [25]–[32]. Other treatments included baclofen [33] and hypnotherapy [34]. Two RCTs included patients with musculoskeletal chest pain (7%, 229 patients): one compared the efficacy of manual therapy to acupuncture and sham intervention [35], while the other compared chiropractic treatment to self-management [36], [37]. The efficacy of treatments based on cognitive behavioral principles was assessed in eleven RCTs (37%, 1370 patients) [38]–[48]. The use of psychotropic drugs compared with placebo was evaluated in seven RCTs (23%, 347 patients) [49]–[55]. Study quality was high in 5 RCTs (17%) and acceptable in 25 (83%; **Table S2**). No studies had to be excluded because of poor study quality. All studies included in the systematic review but not in the meta-analysis were rated to have low (n = 6) to no (n = 3) risk of outcome reporting bias.

**Table 1:** Basis characteristics.

Author, year	Setting	Inclusion criteria	Exclusion criteria	Intervention	Number of patients (women)	Age: years (SD or Range)	Duration of disease: days (SD)
**GI-symptoms**							
Achem, 1997 [25]	Randomized, double-blindtrial of 36 patients referredto the University ofFlorida/Jacksonville forevaluation of the maincomplaint of NCCP duringthe period of January 1992to September 1992, USA	Chest pain retrosternaland suggestive ofcoronary artery disease(squeezing or oppressiveand related to effortand/or emotion) with aduration of at least sixmonths prior to entry anda frequency of at leastthree episodes per week.Patients may have hadother esophagealsymptoms, such aspyrosis or dysphagia, butthe dominant complaint that brought the patient to medical attention was NCCP. GERD was documented by 24-hr ambulatory pH testing. All patients were deemed not to have cardiac disease by a cardiologist (coronary angiography or stress thallium test), all patients had a normal echocardiogram.	Other sources of chest pain: cardiac disease, epicardialcoronary artery disease and valvular heart disease,musculoskeletal causes ofchest pain, reproduction of chest pain on palpation of the chest wall or during arm/neck motion	Omeprazole 20 mgorally twice daily vs.placebo for 8 weeks	36 (23)	52 (N.R.)	<1
Bautista, 2004 [26]	Randomized, double-blind, placebo-controlled, crossover trial of 40 patients who were referred by a cardiologist after a comprehensive evaluation, with at least three episodes per week of unexplained chest pain as the predominant symptom, USA	At least three episodes per week of unexplained chest pain (angina like pain behind the breast bone) for a minimum of 3 months, normal or insignificant findings on coronary angiogram, or had insufficient evidence for ischemic heart disease using exercise treadmill, stress thallium, technetium 99 m tetrofosmin or technetium 99 m methoxy isobutyl isonitrile testing	Cardiac abnormality, severe liver, lung, renal, hematological or any other underlying disorder, previous empirical anti-reflux regimen, history of peptic ulcer disease or gastrointestinal surgery, unwilling or incapable of providing informed consent, and inability to fully complete all phases of the study, duodenal or gastric ulcer as well as other significant lesions on upper endoscopy	Lansoprazole 60 mg AM and 30 mg PM vs. placebo for 7 days	40 (9)	54 (3)	N.R.
Cossentin, 2012 [33]	Randomized, controlled, double-blinded trial with 43 GERD patients with abnormal 24-h pH tests seen in the Gastroenterology Service at the Walter Reed Army Medical Center, 2011, USA	18 years of age or older, have at least one symptom consistent with GERD (to include, but not limited to heartburn, acid taste in the mouth, regurgitation, burning epigastric pain and/or dyspepsia) and evidence of upright (percentage time >6.3) or supine (percentage time >1.2) reflux on 24-h pH testing	Prior gastric or esophageal surgery for the treatment of GERD, severe co-morbid illnesses, history of allergy to baclofen and pregnancy	Baclofen vs. placebo for 14 days	43 (16)	49 (14)	N.R.
Dickman, 2005 [27]	Randomized, double-blind, placebo-controlled, crossover trial of 35 patients with non-cardiac chest pain referred by a cardiologist after a comprehensive cardiac work-up, USA	At least three episodes per week of unexplained chest pain (angina alike pain behind the breast bone) for a minimum of 3 months, normal or insignificant findings on coronary angiogram, or had insufficient evidence for ischemic heart disease using exercise treadmill, stress thallium technetium 99 m tetrofosmin or technetium 99 m methoxy isobutyl isonitrile testing	Severe liver, lung, renal, hematological or any other severe co-morbidity, previous empirical anti-reflux regiment, history of peptic ulcer disease or gastrointestinal surgery, unwilling or incapable of providing informed consent, and inability to fully complete all phases of the study	Rabeprazole 20 mg AM before breakfast and 20 mg PM before dinner vs. placebo for 7 days	35 (12)	56 (10)	N.R.
Dore, 2007 [28]	Randomized controlled study of 266 consecutive patients with heartburn and/or regurgitation, dysphagia and/or odynophagia, with or without esophagitis attending the University Hospital of Sassari, Italy	Heartburn and/or regurgitation, dysphagia and/or odynophagia, with or without esophagitis	Previous endoscopy and or a defined diagnosis of ERD or NERD, upper gastrointestinal (GI) surgery and malignancy, anti-secretory drugs or PPIs during the 4 months before enrollment	Rabeprazole 20 mg vs pantoprazole 20 mg vs esomeprazole 20 mg vs lansoprazole 20 mg for 12 weeks	266 (190)	48 (18–82)	N.R.
Fass, 1998 [29]	Randomized, double-blind, placebo-controlled, crossover trial of 39 patients who were referred by a cardiologist after a comprehensive evaluation, with at least three episodes per week of unexplained chest pain as the predominant symptom, between January and December 1996, USA	At least three episodes per week of unexplained chest pain, insignificant disease or normal anatomy on cardiac angiogram, or lack of evidence of ischemic heart disease on exercise treadmill, stress thallium, technetium 99 m tetrofosmin or technetium 99 m methoxy isobutyl isonitrile testing	Medical contraindication for omeprazole therapy, had already been empirically treated with an anti-reflux regimen, reported a history of peptic ulcer disease or gastrointestinal surgery, or were unwilling or unable to provide informed consent, patients who were unable to fully complete all stages of the study	Omeprazole 40 mg AM and 20 mg PM vs. placebo for 7 days	39 (1)	60 (2)	N.R.
Jones, 2006 [34]	Randomized controlled, single blinded, trial of 28 patients attending the regional cardiothoracic center with angina-like chest pain in whom a coronary angiography was normal, UK	Angina like chest pain in whom coronary angiography was normal, to be able to attend the department over a period of at least 17 weeks, have no coexistent disease, and experience chest pain at least once a week	Esophageal reflux, proton pump inhibitor (it was necessary for them not to have responded to this medication)	4 week baseline period Hypnotherapy: by a therapist, initial tutorial (about condition and factors that may be involved), hypnosis induced by eye closure, followed by progressive muscular relaxation and standard deepening techniques vs. Supportive therapy plus placebo: by a research assistant, counseling and support, placebo medication total 12 sessions over a 17 week period	28 (18)	57	N.R.
Lind, 1997 [30]	Randomized, double-blind study of patients with heartburn, without endoscopic signs of esophagitis, treatment with omeprazole, 20 or 10 mg once daily, or placebo, for 4 weeks (n = 509) at 25 centers in Denmark and Sweden	Both sexes, 18 years or older, with a history of heartburn as the predominant symptom during the past 12 months, with episodes of heartburn occurring on at least 2 days weekly, and without any endoscopic signs of esophagitis	Presence or history of gastric or duodenal peptic ulcer disease or erosive/ulcerative esophagitis, presence of Barrett’s esophagus or an esophageal stricture, previous esophagogastric surgery, treatment with any investigational compound or with anti-secretory agents such as histamine H2-receptor antagonists or PPI in ulcer-healing doses within the month before endoscopy, concurrent cardiovascular, renal, or hepatic disease likely to complicate the evaluation of the study, suspected or confirmed malignancy, clinically significant abnormal findings in the pre-study laboratory screen, or pregnancy or lactation	Omeprazole 20 mg daily vs. 10 mg daily vs. placebo for 4 weeks	509 (303)	50 (N.R.)	N.R.
Pandak, 2002 [31]	Prospective, double-blind, placebo-controlled, parallel, crossover trial of 42 patients who presented with unexplained recurrent chest pain and subsequently had negative results on MIBI testing, ruling out a cardiac etiology, between May 1997 and August 1999, USA.	At least three episodes of chest pain per week of 6 months’ duration or longer	Gastric or duodenal ulcer, were already using anti-reflux medications, had omeprazole contraindications, had prior gastric surgery, or could not comply with the study protocol. Patients with physical exam or chest x-ray abnormalities that would explain their chest pain.	Omeprazole 40 mg orally twice daily vs. placebo for 2 weeks	42 (24)	45 (N.R.)	N.R.
Xia, 2003 [32]	Prospective, single-blind, placebo-controlled trial of 68 patients with chest pain for at least 12 weeks in the preceding 12 months, who were referred to the Cardiology Division of Queen Mary Hospital for evaluation by cardiac catheterization between November 1998 and February 2000, China	Patients who had a normal coronary angiograph, and whose chest pain was considered by a cardiologist to be non-cardiac in origin	Patients with apparent heartburn, acid reflux, dysphagia and dyspepsia	Lansoprazole 30 mg daily vs. placebo for 4 weeks	70 (42)	58 (10)	N.R.
**musculoskeletal**							
Lehtola, 2010 [35]	Randomized, assessor-blinded, placebo-controlled trial of 114 female patients (referred by their GPs) aged 20–60 years with pain in the thoracic spine area during the period of September 1998 to May 2001, Finland	Female subjects, problems in the thoracic area, duration of the symptoms had to be less than 3 months prior to the study	The primary subjective problem was some pain other than thoracic pain (headache, neck pain etc.), a contra-indication to manipulation or acupuncture, and not having experienced pain in the thoracic area within the last 7 days	Facet-traction manipulation: as many segments between the area T3-T8 as necessary, lasted 10–15 min. vs. Acupuncture: points GB21, GV14, GV9–12, BL13–18 la, BL60 la, points GV9–12, lasted 30 min. vs. Placebo: interference-electrotherapy with suction cups without electricity, sucking between 0 and 0.2 bar, lasted 20 min	114 (114)	43 (N.R.)	N.R.
Stochkendahl, 2012 [36], [37]	A non-blinded, randomized controlled trial undertaken at an emergency cardiology department and 4 outpatient chiropractic clinics, 115 consecutive patients with acute chest pain of musculoskeletal origin, between August 2006 and March 2008, Denmark	Aged 18 to 75 years, have had a primary complaint of acute chest pain for less than 7 days duration, should be a resident of the local county, and should be able to read and understand Danish. Participants should have undergone diagnostic procedures to rule out ACS and should not have shown significant comorbidity or contraindications for spinal manipulative therapy.	Previous ACS, prior percutaneous coronary intervention or coronary artery bypass grafting, inflammatory joint disease, insulin-dependent diabetes, fibromyalgia, malignant disease, major osseous anomaly, osteoporosis, apoplexy or dementia, inability to cooperate, and pregnancy	Chiropractic: individual treatment strategy, 1 of 8 experienced chiropractors, high-velocity, low-amplitude manipulation directed toward the thoracic and/or cervical spine, a maximum of 10 treatment sessions of approximately 20 minutes’ duration each, 1 to 3 times per week for 4 weeks vs 15-minute consultation that chest pain generally had a benign, self-limiting course and, based on the clinical evaluation, gave individual instructions regarding posture and 2 to 3 home exercises aimed at increasing spinal movement or muscle stretch	115 (48)	51 (11)	N.R.
**psychiatric drugs**							
Cannon, 1994 [49]	60 consecutive patients with chest pain underwent cardiac, esophageal, psychiatric, and pain-sensitivity testing and then participated in a randomized, double-blind, placebo controlled three week trial of clonidine, imipramine with a morning placebo, or placebo twice daily. This treatment phase was compared with an identical period of twice-daily placebo for all patients (placebo phase), USA	Normal coronary angiograms and no epicardial coronary-artery spasm after the intravenous administration of ergonovine (0.15 mg), normal left ventricular function at rest, no evidence of left ventricular hypertrophy or valvular heart disease (including mitral-valve prolapse) or echocardiography, blood pressure no higher than 160/100 mmHg when they were receiving no medication, and no musculoskeletal sensitivity that accounted for their characteristic chest pain	N.R.	Clonidine 0.05 mg at 9 AM and 9 PM for 1 week, then 0.1 mg for both doses thereafter vs. imipramine 25 mg at 9 PM for 1 week, then 50 mg nightly, always with a matching placebo at 9 AM vs. placebo at 9 AM and 9 PM	60 (40)	50 (29–72)	1590
Cox, 1998 [50]	Randomized, double-blind, cross-over trial of imipramine 50 mg daily vs placebo in 18 women with chest pain and normal coronary angiograms who were suffering at least two anginal episodes per week despite conventional anti-anginal medication, UK	Completely normal coronary angiogram; at least two episodes of anginal pain per week despite anti-anginal medication	Non-cardiac causes of chest pain and patients receiving anti-depressant therapy for previously diagnosed psychiatric disorders	Imipramine 50 mg daily vs. placebo for 5 weeks	18 (18)	53 (35–72)	1500
Doraiswamy, 2006 [51]	Single-site, double-blind, placebo-controlled study of the efficacy and safety of paroxetine in the treatment of chest pain in 50 patients with normal coronary angiograms or stress tests, USA	Males and females between the ages of 18 and 85 experiencing chest pain at minimum 1 to 2 times per week and with a documented normal coronary angiogram or stress test	Treatment with another antidepressant within the 2 weeks (5 weeks for fluoxetine) of beginning double-blind therapy. Medications: narcotics, reserpine, methyldopa, guanethidine, clonidine, local or general anesthetics, and any other psychotropic medication with the exception of hypnotics or benzodiazepines on a minimal, case-by-case basis	Paroxetine 10 mg for 1 week, then increased to 20 mg daily or adjusted to a maximum of 50 mg daily based on clinical response vs. placebo for 8 weeks	50 (21)	53 (N.R.)	1158
Keefe, 2011 [52]	Randomized clinical trial examined the separate and combined effects of CST and antidepressant medication (sertraline) in participants with non-cardiac chest pain. 115 patients diagnosed with NCCP were randomly assigned to one of four treatments: CST plus sertraline, CST plus placebo, sertraline alone, or placebo alone. Participants were recruited from Duke University Medical Center, satellite clinics, and the community from February 2002 to October 2005, USA	Complaints of chest pain in the previous 6 months, received a negative stress test within the past 2 years, normal coronary angiogram within the past 2 years, or had a survival probability >98% at 2 years, a low likelihood of significant coronary artery disease (<25%) on the National Cholesterol Education Program’s (NCEP) modification of the Framingham Risk Calculator (FRC), able to swallow oral medication, and age 18–85 years	Other cardiac problems associated with chest pain, current use of antidepressant medications or medications significantly affecting pain, a history of drug or alcohol abuse within the past 6 months, pregnancy, severe psychopathology (i.e., suicidal patients, severe depression, or psychosis), or treatment with another antidepressant within a period of less than five times the half-life of the drug concerned (e.g. fluoxetine within 5 weeks of beginning double-blind therapy)	Sertraline alone: started at 50 mg daily, adjusted to a maximum of 200 mg within the initial 10 weeks, then dose level stabilized for the remaining 24 weeks vs. CST: 6 sessions, lasted 60 min., bi-weekly for 10 weeks, then 6 follow-up sessions monthly for 6 months with placebo vs. CST with sertraline vs. placebo alone	115 (77)	48 (12)	N.R.
Rao, 2007 [53]	Randomized, controlled, double-blind study with 24 patients with unexplained noncardiac chest pain who were referred to a tertiary care center, USA	At least 12 weeks, which need not be consecutive, in the preceding 12 months of: (a) midline chest pain or discomfort that is not of burning quality and (b) absence of pathologic gastroesophageal reflux, achalasia, or other motility disorder with a recognized pathologic basis. Also, they were required to have a negative cardiac stress test (treadmill exercise stress test, stress thallium test, or stress technetium 99 m-methoxy isobutyl isonitrile [MIBI]) or negative coronary angiography, normal chest X-ray, normal upper GI endoscopy, normal esophageal manometry, and either a normal 24-h pH study (% fraction time of pH <4.0 was <5) or no improvement in chest pain after 2 months of b.i.d. proton pump inhibitor (PPI) therapy	Referred by cardiologists or after a cardiac source for chest pain, muscular-skeletal sources of chest pain, patients who were hospitalized, or those who had significant comorbid illnesses such as cardiac, pulmonary, renal, or hepatic disease or those with diabetes, neuropathy, history of peptic ulcer disease, seizures, or bronchial asthma and those with known allergy or adverse reaction to theophylline, history of psychiatric disorders or who were under treatment with psychotropic drugs	Theophylline oral capsules of 200 mg daily vs. placebo for 4 weeks after meals (crossover: 1 week washout period, then alternate treatment)	24 (16)	44 (22–70)	>84
Varia, 2000 [54]	Single-site, double-blind, placebo-controlled study of the efficacy, tolerability, and safety of sertraline in the treatment of chest pain in 30 outpatients who otherwise had normal coronary angiograms or stress tests, USA	Men and women 18 to 85 years of age who were able to swallow oral medication and who had noncardiac chest pain, negative stress test or normal coronary angiogram. Patients who agreed to abstain from alcohol and to adhere to protocol requirements.	DSM-IV criteria for major depression, panic disorder, or drug or alcohol abuse or dependence. Patients with any active or clinically significant condition, including sensitivity to sertraline, which could possibly affect absorption, distribution, or metabolism of the study drug, Treatment with a depot neuroleptic drug, another antidepressant, fluoxetine, monoamine oxidase inhibitors, reserpine, methyldopa, guanethidine, or clonidine, local or general anesthetic agents, drugs known to interact with sertraline, antidepressants, other psychotropic medications, or medications significantly affecting pain	Sertraline started at 50 mg daily, adjusted to a maximum of 200 mg vs. placebo for 8 weeks	30 (N.R.)	N.R.	N.R.
Wulsin, 2002 [55]	Randomized, controlled, non-blind, trial of a protocol intervention to initiate panic disorder treatment of 156 enrolled participants, all at low to moderate risk for acute coronary syndrome, admitted to the University Hospital Chest Pain Center in the ED during a 14- month period (March 1998 to May 1999), USA	All adult (≥18 years)	Cognitive impairment, active severe substance abuse, and bipolar disorder	Intervention: patient education by the research assistant about panic disorder according to study protocol, initiation of treatment with a 1-month supply of paroxetine 20 mg/d vs. Usual care: consisted of reassurance that the patient has no cardiac disease, care as needed through primary care physician, telephone follow-up at 3 months	50 (35)	43 (N.R.)	N.R.
**psychological interventions**							
Arnold, 2009 [38]	Single center, non-blinded, randomized controlled trial of 700 consecutive patients with acute chest pain and no clear diagnosis at initial presentation referred to the chest pain unit of an emergency department between May 2006 and September 2007, UK	Chest pain of possible cardiac origin, were aged over 25, had no changes for acute coronary syndrome on a diagnostic electrocardiogram	Suspected life threatening non-cardiac disease, known coronary heart disease presenting with recurrent or prolonged episodes of cardiac-type chest pain	Standard verbal advice or verbal advice followed by an information sheet	700 (269)	49 (12)	N.R.
Esler, 2003 [39]	Randomized controlled, non-blinded, trial with 59 patients who presented to a large, university-affiliated Level One Trauma Center ED with chief complaints of chest pain and were determined to have NCCR, USA	No known history or suspected history of coronary artery disease, other significant medical illness (e.g. congestive heart failure, pulmonary embolism, or lung disease), and no obvious proximal cause of their chest pain (e.g. pneumonia, bronchitis, or chest trauma)	Current psychosis, suicidal or homicidal ideation, and those who were intoxicated from alcohol or other drugs at the time of admission to the ED	CBT (cognitive behavioral treatment) intervention (involving psycho education, diaphragmatic breathing exercises, and cognitive restructuring about physical symptoms), 60 min, 1 psychologist vs. treatment-as-usual	59 (27)	41 (12)	<30: 45.8%, 30–180: 15.3%, 180–720: 5.1%, >720: 33.9%
Gasiorowska, 2008 [56]	A randomized, controlled pilot study of 39 patients with at least three episodes per week of unexplained chest pain for 3 consecutive months, Gastroenterology Service, University of Arizona Health Sciences Center, USA	at least three episodes per week of unexplained chest pain for 3 consecutive months, either insignificant coronary artery disease, normal coronary arteries on cardiac angiogram or lack of evidence of ischaemic heart disease on an exercise treadmill, stress thallium, technetium 99 m tetrofosmin or technetium 99 m sestamibi testing, normal upper endoscopy, pH testing and esophageal manometry	severe underlying comorbidities, upper airway symptoms such as hoarseness, wheezing and laryngospasm, diabetes mellitus, scleroderma, gastroparesis, peptic ulcer disease, history of gastrointestinal surgery, depression, autonomic or peripheral neuropathy or neuromuscular disorder, patients using narcotics, benzodiazepines, tricyclic antidepressants or selective serotonin reuptake inhibitors, patients unable to complete the upper endoscopy, 24-hour esophageal pH monitoring or esophageal manometry, patients demonstrating erosive esophagitis, Barrett’s esophagus or other GERD-related complications during upper endoscopy, abnormal pH test or manometry results	Johrei treatment (process of transmission of healing energy) delivered by an experienced and certified Johrei practitioner in a hospital clinic with minimal interaction with the patient. Each Johrei treatment session usually lasts 20 min. vs. waiting list	39 (13)	54.5 (11.9)	N.R.
Hess, 2012 [40]	Randomized controlled, single blinded, trial of 204 patients attending the ED of Saint Mary’sHospital at the Mayo Clinic with symptoms suggestive of ACS, USA	Aged >17 years who presented to the ED with primary symptoms of non-traumatic chest pain and who were being considered for admission to the ED observation unit for monitoring and cardiac stress testing within 24 hours	Elevated initial cardiac troponin T levels above the 99th percentile reference limit (99th percentile, <0.01 ng/mL; lower limit of detection, 0.01 ng/mL; 10% coefficient of variation, 0.035 ng/mL), known coronary artery disease (defined as 1 50% stenosis on cardiac catheterization; prior electrocardiographic changes indicative of ischemia, e.g. ST-segment depression, T-wave inversion, or left bundle branch block, perfusion defects or wall motion abnormalities on previous exercise, pharmacological, or rest imaging studies; previous documentation of acute myocardial infarction, or if no records were available, patient self-report of coronary artery disease), cocaine use within the previous 72 hours by clinician history, or pregnancy	Decision aid (information paper): included a 100-person pictograph depicting the pretest probability of acute coronary syndrome and available management options (observation unit admission and stress testing or 24–72 hours outpatient follow-up) after initial evaluation (ECG, interpretation, results of initial cardiac troponin testing, plan for serial cardiac markers) vs. usual care	204 (120)	54.7 (11.9)	0.5 (2)
Jonsbu, 2011 [41]	A randomized controlled, non-blinded, trial of 40 patients with persistent complaints six months after a negative evaluation at a cardiological outpatient clinic between May 2006 and May 2007 at Molde Hospital, Norway	Aged between 18 and 65 years, persistent symptoms or limitations in activity six months after the cardiac evaluation: at least weekly symptoms of chest pain or palpitations, at least “some impact” on family life, social life, or work from the symptoms, at least “rare but sometimes” avoidance of physical activity because of worry about the heart	Cardiac disease in need of treatment	Intervention: cognitive behavioral treatment (CBT), 3 sessions, lasted 60–90 min., at the Psychiatric Outpatient Clinic at Molde Hospital vs. Control group: usual care from their general practitioner, free to use the health care system when needed	40 (26)	52 (22–65)	360
Lahmann, 2008 [42]	A randomized, controlled, clinical investigation of 22 patients presenting with non-specific chest pain at the University Hospital of Regensburg, Germany	Over the age of 18 years who presented with NSCP	Any underlying somatic disorders; any severe and disabling psychiatric disorder, such as schizophrenia or dementia; as well as patients undergoing psychotherapy and those current enrolled for retirement payment	Functional relaxation: started with a 60-minute psychosomatic-education session, then 10 group-therapy sessions (each 90 min) during 6 weeks vs. enhanced medical care: treatment-as-usual plus 2 case-management counseling interviews (personal-care skills, shared decision-making)	22 (12)	44.35	1773 (2218.5)
Mayou, 1997 [44]	Randomized controlled, non-blinded, trial of 37 patients presenting with chest pain and reassured by a cardiologist they do not have heart disease, UK	Aged 18–65, presence of persisting non-cardiac chest pain occurring at least once a week in the month before the assessment	Cardiac diagnosis, current major depression, living outside the country and being unable to speak English	Cognitive behavioral treatment: consisted of up to 12 sessions of individual therapy with a research counseling psychologist (D.S.) trained and supervised by a clinical psychologist (I.K.) vs. usual care	37 (22)	49 (N.R.)	<180: 11, 180–720: 12, >720: 11
Mayou, 2002 [43]	A randomized controlled, single-blind, trial within a cardiology clinic (referred from primary care practice) at a district general hospital, 80 consecutive patients diagnosed as having benign palpitation ± either palpitation due to awareness of extrasystoles or sinus rhythm ± with associated distress or disability, UK	Aged 17 or over referred to a district cardiac clinic for the assessment of palpitation, suffering from benign palpitation and who described distress or disability	Subjects with very short histories or who did not describe distress or disability	Intervention group: usual care plus nurse-delivered intervention based on cognitive-behavioral principles coordinated with cardiological care, derived from experience with the early treatment of NCCP vs. Control group: usual cardiological care, with no contact with a cardiac nurse either in out-patients or by telephone	80 (57)	44 (N.R.)	N.R.
Potts, 1999 [45]	Randomized, non-blinded, trial of 60 patients who had continuing chest pain despite cardiological reassurance following haemodynamically normal angiography in the Departments of Cardiology of the Royal Infirmary and Western General Hospitals in Edinburgh, Scotland	Aged 18–70, recent (within the last year) coronary angiography for the investigation of chest pain revealed coronary arteries which were either normal or <50% stenosed, chest pain continuing at least twice weekly after angiography, despite reassurance by the cardiology team, and residence within easy travelling distance of Edinburgh	Past history of myocardial infarction, or serious concurrent physical or psychiatric illness	Intervention (CBT): small groups with a maximum of six subjects. Groups met weekly for 4 weeks, then every two weeks for a further 4 weeks. Each session lasted 2 h	60 (36)	54 (N.R.)	
Sanders, 1997 [46]	Randomized controlled, single-blind, study of 57 consecutive patients with chest pain and normal angiograms over a 12-month period, UK	Normal coronary arteries, not need further medical investigations	All those who were either admitted to a Day Case Unit, and went home once they had received the results of the angiogram, or else stayed overnight on the cardiac ward	1-hour intervention: individualized information and discussion session by a specially trained cardiac nurse, together with a handout and cassette providing information and advice and telephone follow-up to discuss progress, answer questions and reiterate advice vs. usual care	57 (36)	N.R.	<180: 16, <720: 21, >720: 19
Van Peski, 1999 [48]	Randomized controlled, non-blinded, trial of 72 patients presenting with chest pain referred by their general practitioners to the cardiology clinic of the Leiden University Hospital or the Diaconessenhuis Hospital between 1992 and the first months of 1996 and who had received a discharge diagnosis of NCCP, Netherlands	18 to 75 years old with chest pain as the main complaint, who had a normal cardiovascular system according to a cardiologist	Proven coronary artery disease or myocardial ischemia, as demonstrated by coronary angiography, exercise testing, laboratory examination, electrocardiogram, or chest x-ray, a history of typical angina pectoris; insufficient fluency in Dutch, current psychiatric treatment for NCCP, current diagnosis of an organic mental syndrome, psychotic disorder; major depression, bipolar disorder, or use of psychoactive substances (excluding nicotine) within 3 months before study entry	Cognitive behavioral treatment: 4 to 12 weekly sessions of 45 to 60 minutes, depending on the severity of the patient’s problem, the maximum duration of therapy was 6 months. Treatment was modeled after cognitive-behavioral therapy for panic, hypochondriasis, and unexplained physical symptoms, each patient received written information about the therapy model and treatment procedures. The therapists were a physician with basic training in cognitive-behavioral therapy and a senior psychologist trained in cognitive-behavioral therapy vs. Usual care: free to use health resources as they saw fit. They were seen in the cardiology clinic for the follow-up assessments 6 months and 12 months after the baseline interview	72 (36)	49 (11)	1500 (1770)

ACS, acute coronary syndrome; CBT, cognitive behavioral therapy; CST, coping skills training; ECG, electrocardiogram; GERD, gastroesophageal reflux disease; GI, gastro-intestinal; mg, milligram; MIBI, methoxy-isobutyl-isonitril; min, minutes; n.r., not reported; NCCP, non-cardiac chest pain; NSAID, nonsteroidal antiinflammatory drugs; PPI, proton-pump inhibitor; vs., versus.

### Treatment efficacy for NCCP due to gastroesophageal reflux disease (GERD)

**Figure 2:**
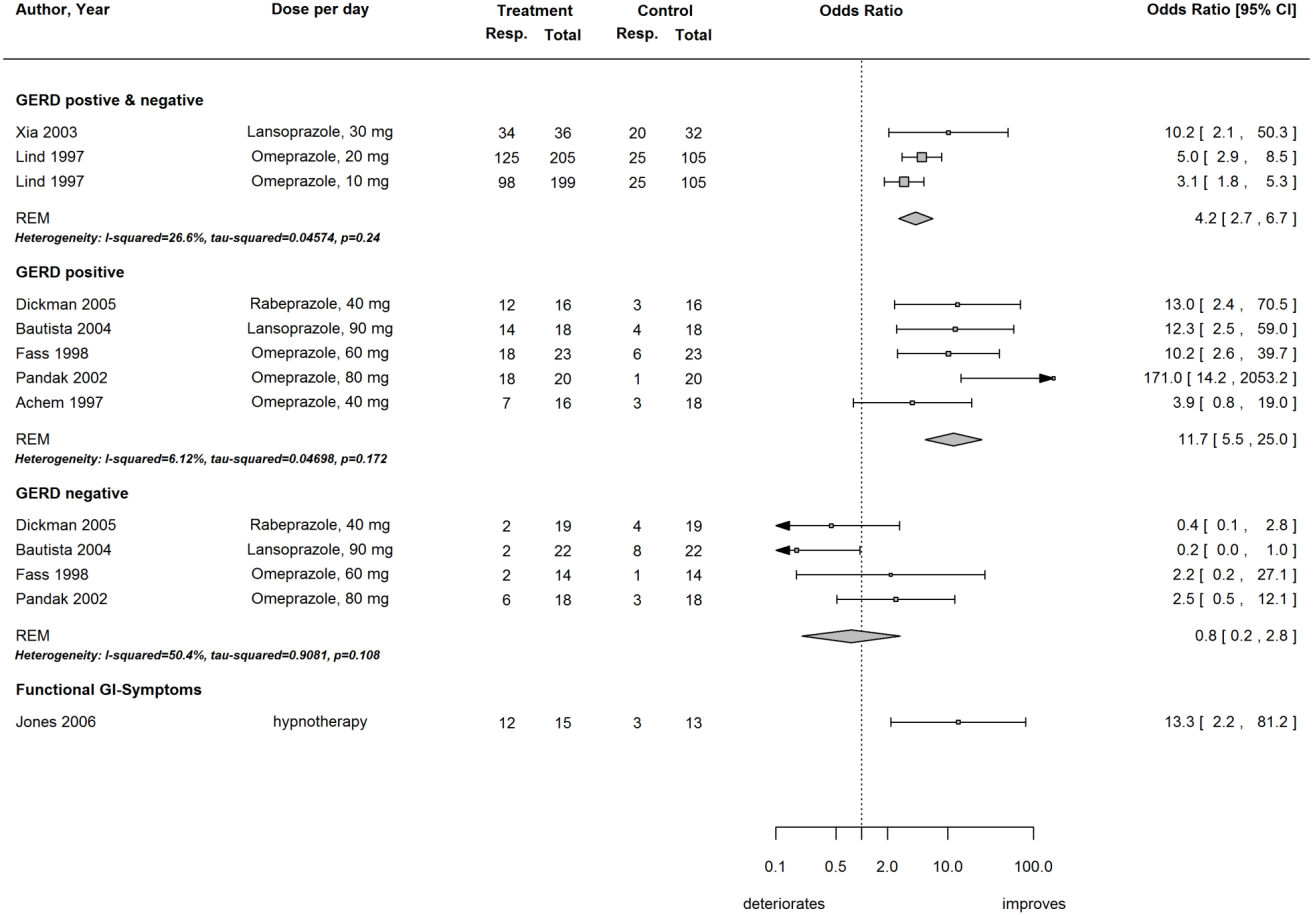
Efficacy of Proton Pump Inhibitor (PPI) treatment in patients with NCCP.

Only similar RCTs (n = 7, 771 patients) were included in the meta-analysis (Figure 2). A PPI was administered in most RCTs twice daily for 1 to 8 weeks. In two RCTs, GERD-positive and GERD-negative patients were not distinguished [30], [32]; in both of these studies, the efficacy of treatment was more effective than placebo. The pooled odds ratio for a reduction in chest pain of ≥50% was 4.2 (95% CI 2.7 to 6.7, heterogeneity I^2^ = 26.6%).

When patients with GERD [n = 5, 192 patients, GERD-positive: confirmed by upper endoscopy and/or 24-h pH manometry [25]–[27], [29], [31]] were compared with patients without GERD [n = 4, 156 patients, GERD-negative [26], [27], [29], [31]] the treatment efficacy of the PPI treatment increased. The pooled OR for GERD-positive patients with NCCP was 11.7 (95% CI 5.5 to 25.0, heterogeneity I^2^ = 6.1%); in comparison, the pooled OR for patients without GERD was 0.8 (95% CI 0.2 to 2.8, heterogeneity I^2^ = 50.4%).

**Figure 3:**
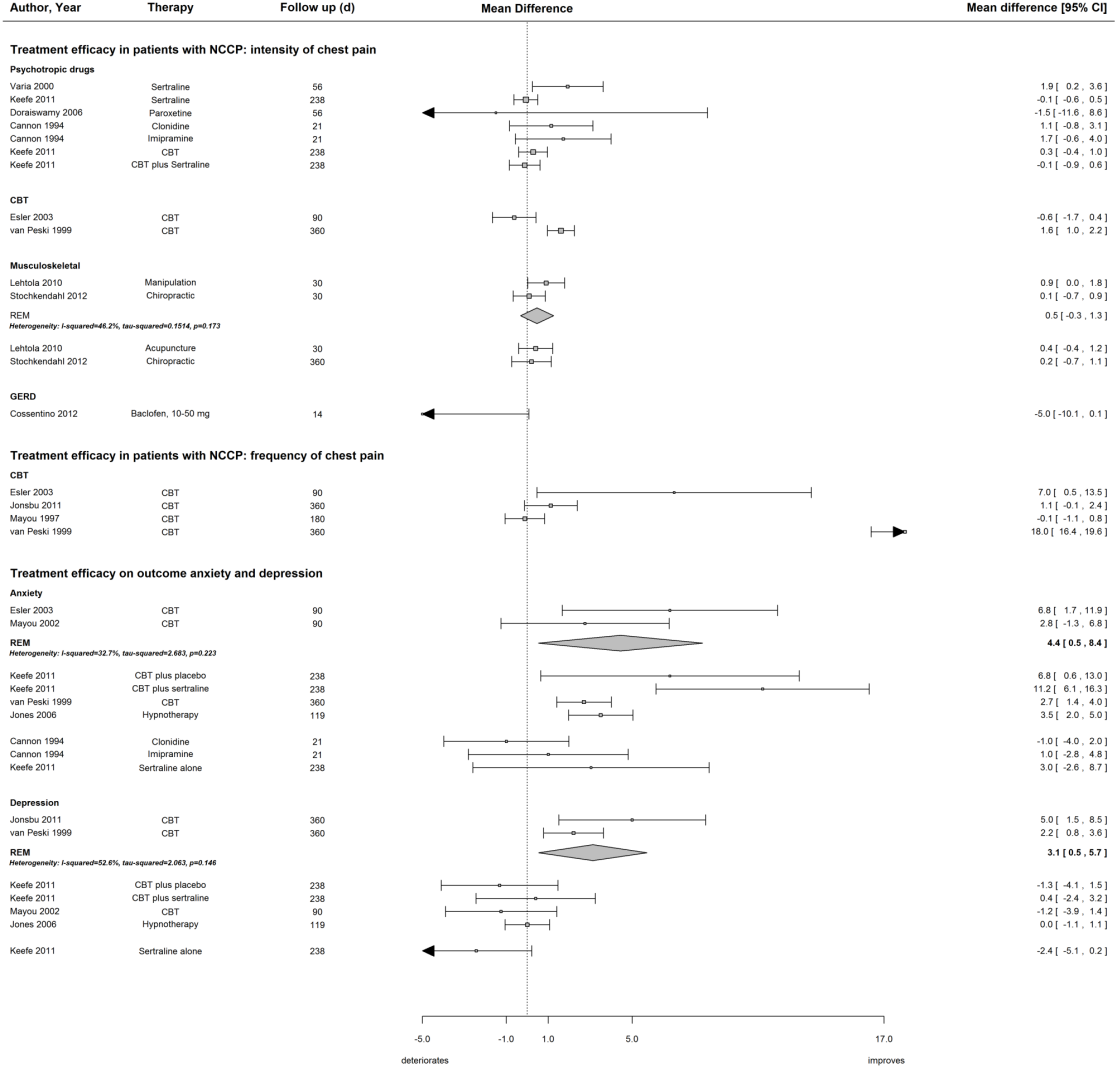
Treatment efficacy in patients with NCCP: intensity and frequency of chest pain.

While the heterogeneity among GERD-positive patients was low and three of five RCTs emanated from the same center [26], [27], [29], there was some heterogeneity among the GERD-negative patients. Regarding GERD-negative patients, two RCTs found a trend toward more chest pain in the treatment group [26], [27] while two RCTs found a trend toward less chest pain in the PPI group [29], [31]. Funnel plots for the three groups (GERD-positive and GERD-negative mixed, GERD-positive alone, and GERD-negative alone) are depicted in **Figure S1**. There was no evidence for funnel plot asymmetry in the mixed group (GERD-positive and GERD-negative, p = 0.27) or in the GERD-negative group (p = 0.68); however, some evidence of funnel plot asymmetry was observed in the GERD-positive group (p = 0.04), as assessed using Egger’s regression test [22]. We refrained from statistical adjustment for outcome reporting bias as proposed by Copas et al. [19] because only one PPI treatment study was not included in our meta-analysis [28]. Dore et al. did not analyze the outcome of interest in our study and therefore the study was associated with a low risk of outcome reporting bias (**Table S3**). Jones et al. investigated the efficacy of hypnotherapy in patients with functional gastrointestinal symptoms (Figure 2) [34]. Baclofen treatment was associated with more chest pain (Figure 3) at the 2-week follow-up [33]. Detailed results for all studies are summarized in **Table S4**.

### Treatment efficacy regarding the intensity and frequency of non-cardiac chest pain

Treating patients with NCCP without a specific diagnosis of psychiatric disease with the selective serotonin reuptake inhibitor sertraline was more effective after 2 months [54], but not after 8 months of follow-up for chest pain intensity (Figure 3) [52]. Although use of the tricyclic antidepressant imipramine exhibited a trend towards less chest pain intensity, the effect was not statistically significant [49]. Findings were mixed regarding treatments based on the principles of cognitive behavioral therapy (CBT). One study showed that CBT (4 to 12 60-min sessions) more effectively reduced chest pain intensity at 1 year than usual care at the cardiology department [48]. Another study found that cognitive skill training (5 bi-weekly and 6 30-min monthly sessions), alone or supplemented with sertraline, was no more effective than placebo or sertraline alone in reducing chest pain intensity at 8 months of follow-up [52]. A brief CBT intervention was not more effective in reducing chest pain intensity (Figure 3), but more effectively reduced chest pain frequency (Figure 3) at 3 months of follow-up [39]. Two smaller RCTs found no [44] or only a trend towards [41] less frequent chest pain after CBT interventions (Figure 3). In a small pilot study Johrei, a process of transmission of healing energy, was compared to a waiting list [56]. While the treatment group experienced a significant improvement in symptom intensity these findings need further validation. The heterogeneity of the study settings and treatments used prevented us from conducting a meta-analysis.

One study of patients with musculoskeletal NCCP found that manipulation reduced pain more effectively than acupuncture and usual care (Figure 3) [35]. Another study found that chiropractic treatment and home exercise were similarly effective [36]. The pooled mean difference for the manipulation therapy was 0.5 (95% CI −0.3 to 1.3, heterogeneity I^2^ = 46.2%).

### Efficacy of treatment regarding anxiety and depression

Only similar studies were included in meta-analyses. CBT more effectively reduced anxiety scores in all studies (Figure 3). The pooled mean difference for CBT in two similar studies [39], [43] was 4.4 (95% CI 0.5 to 8.4, heterogeneity I^2^ = 32.7%).

CBT treatment reduced depression scores more effectively than usual general practitioner (GP) care after 1 year [41], [47]. The pooled mean difference for CBT was 3.1 (95% CI 0.5 to 5.7, heterogeneity I^2^ = 52.6%). A study with four arms compared CBT or no treatment and sertraline or placebo [52]. While CBT with and without sertraline was more effective at reducing anxiety scores, they were no more effective at reducing depression scores than placebo. At baseline, depression scores were lower than anxiety scores in this study population (depression 8.9–10.2, range 0–63; anxiety 34–41, range 20–80).

## Discussion

### Main findings

The systematic analysis of 30 RCTs involving patients with NCCP demonstrated that PPI treatment was effective in patients with GERD. In NCCP patients without GERD, PPI treatment was no more effective than placebo. Treatment based on cognitive behavioral principles reduced chest pain frequency compared with ‘general practitioner treatment’ in three RCTs, while one study found no effect. Most studies that compared the efficacy of serotonin antagonists or tricyclic agents with placebo demonstrated no difference or only a trend towards less pain intensity in the treatment arms. Manipulative treatment interventions produced conflicting results for patients with musculoskeletal chest pain, and acupuncture was no more effective than usual care. For most prevalent diseases that manifested with chest pain, only a few studies were available.

### Results in light of the existing literature

To our knowledge, this is the first comprehensive systematic review and meta-analysis to summarize the current evidence on treatment efficacy based on RCTs for various diseases presenting in patients with NCCP. Recently new therapies for NCCP of gastrointestinal origin were discussed [57]. Treatment interventions including nitrates, Phosphodiesterase-5 inhibitors, anticholinergics, calcium channel blockers, and endoscopic injection of botulinum toxin may be effective in a subset of patients with gastrointestinal diseases. Most of these interventions have been studied in non-randomized trials or case series [58]. This systematic review and meta-analysis confirms that limited RCTs are available for many interventions and highlights the need for further studies. Non-randomized trials tend to overestimate treatment effects [59]. Further, this comprehensive overview addresses the need for interdisciplinary evaluation and care in patients with NCCP and summarizes the evidence for treatment interventions in underlying diseases oftentimes not considered. A recent systematic review found evidence that NCCP patients have similar levels of psychological morbidity than patients with cardiac chest pain and higher levels than healthy controls [60]. While gastroesophageal diseases are common these findings indicate that other diseases might not be diagnosed.

Only limited evidence was available for most prevalent diseases that manifest with chest pain. Only two RCTs investigated treatment efficacy of manual therapy in patients with musculoskeletal chest pain [35], [36]. The efficacy of psychotropic drugs on chest pain intensity, anxiety and depression scores were in line with a recently published meta-analysis that analyzed the efficacy of CBT compared with pharmacotherapy in adults with major depressive disorder (21 RCTs, 2027 patients) or anxiety disorder (21 RCTs, 1266 patients) [61]. The authors found CBT to be equally effective as pharmacotherapy in patients with depression whereas CBT was somewhat more effective than pharmacotherapy in anxiety disorders [61]. It has been shown that patients discharged from the emergency department with the diagnosis of NCCP had elevated anxiety levels compared to healthy individuals [62]. Anxiety disorder might be an underlying disease for subjects with chest symptoms to seek evaluation in emergency departments. Interestingly, CBT was more effective in patients with panic disorders [61]. This information may be relevant for further management of patients with unexplained chest pain. No study was identified that investigated panic disorders in patients with NCCP. However, in patients that present with NCCP to the emergency department, panic disorders are often not diagnosed [63], [64].

A recent meta-analysis using a hierarchical Bayesian model demonstrated the diagnostic value of the response to high-dose PPI treatment in patients with GERD [posterior mean sensitivity 0.89 and specificity of 0.88 [16]]. Together with the current findings in patients with NCCP, in which GERD is suspected, PPI treatment should be initiated early and PPI treatment response should be evaluated after 2 weeks [16]. While the findings are in line with previous published meta-analyses on PPI treatment studies [65], [66], this is the first meta-analysis to assess study quality and the risk for outcome reporting bias. In comparison to Kahrilas et al. [65], [66] and Cremonini et al. [65], [66], one additional study was included [30]. Further, Cremonini et al. [65], [66] included open-label studies and did not distinguish between GERD-positive and GERD-negative patients. We also used odds ratios as the effect measure for pooling because of its favorable mathematical properties over the relative risk. This includes that the odds ratio is unbounded regardless of the underlying event rate [67]. Our study expands on a relevant aspect in the clinical setting where the PPI response is often used for the diagnosis of GERD. We showed that the effect in a mixed patient sample is smaller compared to patients where GERD was diagnosed by a reference test (e.g. 24-hour pH monitoring). Therefore, a lack of response to PPI treatment after 2 weeks should lead to discontinuation of PPI treatment, while a response indicates underlying GERD for which PPI treatment is effective.

### Strengths and limitations

This review comprehensively evaluates the currently available studies. The search was inclusive; no language restrictions were applied, a thorough bibliographic search was conducted to identify all relevant studies, and rigorous methodology was applied. The extraction process was performed in accordance with current guidelines and supported by an experienced statistician. Potential factors influencing treatment efficacy were identified by a multidisciplinary team (an internist, general practitioner, statistician, and methodologist).

The main limitation of this systematic review and meta-analysis was the limited number of RCTs. Many interventions used in clinical practice in patients with NCCP were not assessed in RCTs. Of the included studies, many were only of moderate methodological quality. Furthermore, NCCP is a collective term with potentially different underlying diseases and therefore might present differently. Treatment efficacy in one population in which the prevalence for one disease is high might be entirely different for another population [68]. In addition, the heterogeneity of the outcome measures used and follow-up durations reported prevented us from including most studies in our meta-analysis. The results of these studies should be interpreted on an individual study level within the context of the study population. We have tried to balance this by providing a thorough description of the studies inclusion and exclusion criteria and the study settings. This information will allow readers to judge to whom various study results apply.

### Research implications

Additional research should compare diagnostic indicators (e.g., pain increase with movement or decrease on medication were associated with musculoskeletal chest pain) [16] plus a corresponding treatment intervention to usual care alone in defined patient populations (e.g., emergency departments, primary care). Future research should also aim to contribute to our knowledge about diagnostic processes and treatment decisions for patients with NCCP. Although most patients with chest pain consult primary care physicians [69], few studies are performed in this setting. Additional research is needed to strengthen the evidence in a primary care setting. Screening questionnaires for panic and anxiety disorders could be used to identify patients that need further specialized assessment and would respond well to treatments based on cognitive behavioral principles. No such study was found in the current analysis.

### Implication for practice

Patients with NCCP incur high healthcare costs owing to extensive and often invasive diagnostic testing, as well as the effect of NCCP on quality of life. Early identification of underlying diseases is essential to avoid delayed treatment and chronicity of complaints. Symptoms and clinical findings may provide important information to guide treatment of an underlying illness [16]. In patients with typical GERD symptoms, twice-daily high-dose PPI treatment is the most efficient diagnostic and therapeutic approach. GERD is very likely if a positive treatment response occurs after 1 week, and is unlikely if there is no response after 4 weeks of PPI treatment [16]. In patients that do not respond to PPI, PPI treatment should be stopped if an endoscopy reveals no pathological findings.

Panic and anxiety disorders are often missed in clinical practice [70]. For patients with anxiety, treatments based on cognitive behavioral principles might be more effective than pharmacologic treatment. To date, evidence for the efficacy of serotonin antagonists or tricyclic agents in patients with NCCP is weak.

## Conclusion

Timely diagnostic evaluation and treatment of the underlying disease is important for patients with NCCP. The current systematic review and meta-analysis showed a lack of RCTs for many diseases presenting with NCCP or treatment interventions proposed in the literature. Only limited evidence was available for prevalent diseases that manifest with chest pain. In addition, many treatment interventions that have been shown to be effective in non-randomized trials have not been studied in RCTs and might overestimate treatment efficacy. In patients suspected to have GERD high-dose treatment with a PPI is effective. In otherwise unexplained NCCP treatments based on cognitive behavioral principles might be considered. There is a need for further high quality studies addressing the gaps highlighted in this review.

## Supporting Information

Figure S1 **Risk of bias assessment by using Funnel Plot.**
(TIF)

Table S1 **Search Strategy July Week 28, 2013.**
(DOCX)

Table S2 **Summary of the SIGN quality assessment.**
(DOCX)

Table S3 **Risk for Outcome Reporting Bias in Trials (ORBIT) in studies excluded from Meta-analyses.**
(DOCX)

Table S4 **Detailed summary of results.**
(DOCX)

Checklist S1 **PRISMA checklist.**
(DOC)
