# Supplementary figures and images for: Treatment Efficacy for Non-Cardiovascular Chest Pain: A Systematic Review and Meta-Analysis
Source: PLoS One. 2014 Aug 11;9(8):e104722. doi: 10.1371/journal.pone.0104722 (PMC4128723; doi:10.1371/journal.pone.0104722)

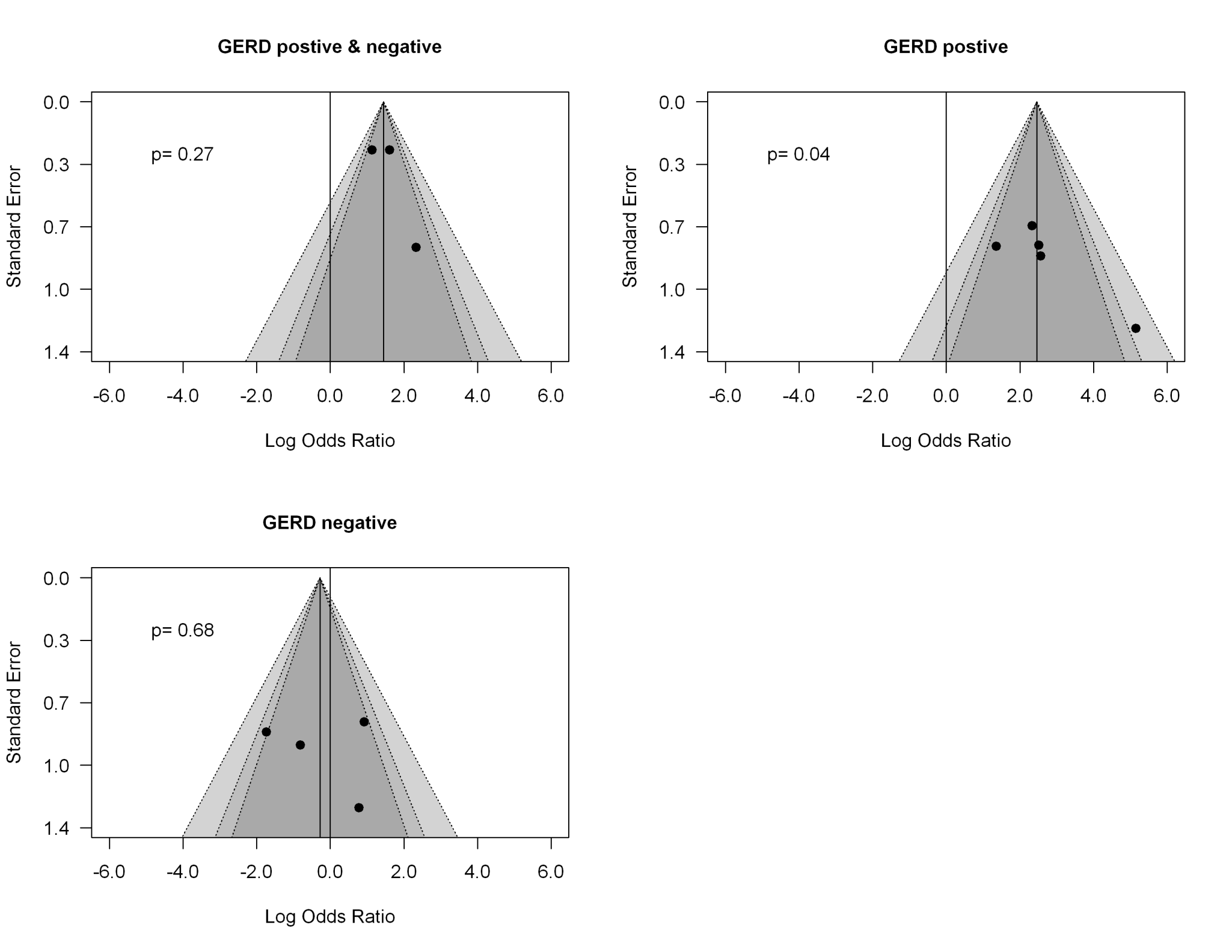

Supplement: Figure S1 — Risk of bias assessment by using Funnel Plot. (TIF) [file pone.0104722.s001.tif]
