# Supplementary material for: Treatment Efficacy for Non-Cardiovascular Chest Pain: A Systematic Review and Meta-Analysis
Source: PLoS One. 2014 Aug 11;9(8):e104722. doi: 10.1371/journal.pone.0104722 (PMC4128723; doi:10.1371/journal.pone.0104722)
Supplement: Table S1 — Search Strategy July Week 28, 2013. (DOCX) [file pone.0104722.s002.docx]

Table S1: Search Strategy July Week 28, 2013

1a) Ovid MEDLINE(R), Ovid MEDLINE(R) In-Process & Other Non-Indexed Citations, Ovid MEDLINE(R) Daily and Ovid OLDMEDLINE(R) 1946 to Present

| # |  | Results |
| --- | --- | --- |
| 1 | ((chest or thorax or thoracic) adj3 (pain or burn)).tw. | 25208 |
| 2 | thoracic spine pain.tw. | 27 |
| 3 | exp Chest Pain/ | 52813 |
| 4 | 1 or 2 or 3 | 69056 |
| 5 | ((missing or lacking) adj5 diagno*).tw. | 1223 |
| 6 | (("not yet diagnosed" or "not diagnosed" or "no diagnose" or "without diagnose") adj10 "chest pain").tw. | 6 |
| 7 | ((noncardiac or non-cardiac or atypical or musculoskeletal or ?esophageal or enigmatic* or mysterious or puzzling or cryptic* or unkown or not-known or non-specific or "non specific" or vague or undefined or undetermined or unspecific or indefinite or ambiguous or unclear or indistinct or indistinctive or obscure or blurry) adj10 "chest pain").tw. | 2433 |
| 8 | or/5-7 | 3660 |
| 9 | 4 and 8 | 2451 |
| 10 | exp Hospitalization/ or exp Emergency Service, Hospital/ or exp "Length of Stay"/ | 194983 |
| 11 | exp Patient Readmission/ | 7583 |
| 12 | exp Patient Discharge/ | 18502 |
| 13 | (ecu visit* or re-admi* or readmi* or re-hospital* or rehospital* or previous* hospitali?* or (health adj3 (care or service*) adj3 (consum* or seeking behavio?r* or use* or utili?ation*)) or (repeat* adj3 visit*) or ((subsequent* or recurrent or repeat* or previous*) adj3 hospital adj2 (admission* or encounter* or visit*)) or (patient* adj2 discharge*)).tw. | 63847 |
| 14 | exp Patient Discharge/ | 18502 |
| 15 | or/10-14 | 18502 |
| 16 | 4 and 15 | 242357 |
| 17 | 9 or 16 | 4624 |
| 18 | Cross-Sectional Studies/ or case reports/ or Comment/ or Editorial/ or Letter/ or "review"/ | 4692349 |
| 19 | 17 not 18 | 4865 |
| 20 | incidence/ or exp mortality/ | 438374 |
| 21 | Follow-Up Studies/ | 489259 |
| 22 | (prognos* or predict* or course*).tw. | 1620315 |
| 23 | exp Prognosis/ | 1077326 |
| 24 | Morbidity/ | 23148 |
| 25 | mortality.fs. | 414202 |
| 26 | exp Survival Rate/ | 125854 |
| 27 | "Outcome Assessment (Health Care)"/ | 48922 |
| 28 | exp treatment outcome/ | 642883 |
| 29 | ((disease or disorder) adj3 (incidence or frequency)).tw. | 19983 |
| 30 | (morbidity or mortality).tw. | 558657 |
| 31 | (death or exitus).tw. | 465060 |
| 32 | (relaps* or recrudescence or deteriorat* or exacerbat* or worse* or recover* or recurren*).tw. | 1131049 |
| 33 | (Cure* or curative* or resolv* or resolution* or heal* or improv* or recuperat* or survival).tw. | 3741815 |
| 34 | or/20-33 | 6677911 |
| 35 | 19 and 34 | 3588 |
| 36 | exp musculoskeletal diseases/ or exp digestive system diseases/ or exp respiratory tract diseases/ or exp cardiovascular diseases/ | 4704308 |
| 37 | exp Mental Disorders/ | 943388 |
| 38 | ((heart or cardiac or cardiovascular or vascular or coronary or thoracic or thorax or pulmonary or lung or bronchial or musculoskeletal or digestive or gastrointestinal or mental or psycho*) adj3 (disease* or disorder*)).tw. | 483834 |
| 39 | (heart failure or stroke or cox).tw. | 314091 |
| 40 | or/36-39 | 5746510 |
| 41 | 35 and 40 | 3272 |
| 42 | limit 41 to "all child (0 to 18 years)" | 228 |
| 43 | limit 42 to "all adult (19 plus years)" | 185 |
| 44 | 42 not 43 | 43 |
| 45 | 41 not 44 | 3229 |
| 46 | limit 45 to animals | 5 |
| 47 | limit 46 to humans | 5 |
| 48 | limit 45 to yr="1993 -Current" | 2772 |

1 b) Database: Embase ([www.embase.com](http://www.embase.com))

| 1 | 'noncardiac chest pain'/exp OR 'musculoskeletal chest pain'/exp | 502 |
| --- | --- | --- |
| 2 | ((noncardiac OR 'non cardiac' OR musculoskeletal OR ?esophageal) NEAR/3 'chest pain'):ab,ti | 1,471 |
| 3 | #1.1 OR #1.2 | 1,686 |
| 4 | 'thorax pain'/exp | 43,078 |
| 5 | ((chest OR thorax OR thoracic) NEAR/3 (pain OR burn)):ab,ti | 34,221 |
| 6 | #1.4 OR #1.5 | 56,943 |
| 7 | ((missing OR lacking) NEAR/5 diagno*):ab,ti | 1,637 |
| 8 | (('not yet diagnosed' OR 'not diagnosed' OR 'no diagnose' OR 'without diagnose') NEAR/10 'chest pain'):ab,ti | 9 |
| 9 | ((noncardiac OR 'non cardiac' OR atypical OR musculoskeletal OR ?esophageal OR enigmatic* OR mysterious OR puzzling OR cryptic* OR unkown OR 'not known' OR 'non specific' OR 'non specific' OR vague OR undefined OR undetermined OR unspecific OR indefinite OR ambiguous OR unclear OR indistinct OR indistinctive OR obscure OR blurry) NEAR/10 'chest pain'):ab,ti | 3,136 |
| 10 | #1.7 OR #1.8 OR #1.9 | 4,779 |
| 11 | #1.6 AND #1.10 AND [1993-2005]/py | 1,110 |
| 12 | 'hospitalization'/exp OR 'emergency health service'/exp OR 'hospital readmission'/exp OR 'hospital discharge'/exp | 303,565 |
| 13 | readmi*:ab,ti OR rehospital*:ab,ti | 21,918 |
| 14 | 're admission':ab,ti OR 're hospitalization':ab,ti | 2,053 |
| 15 | #1.12 OR #1.13 OR #1.14 | 313,335 |
| 16 | #1.6 AND #1.15 AND [1993-2005]/py | 1,388 |
| 17 | #1.3 OR #1.11 OR #1.16 | 3,534 |
| 18 | 'case study'/exp OR 'case report'/exp OR 'cross-sectional study'/exp OR 'letter'/exp OR 'editorial'/exp OR 'review'/exp | 5,061,838 |
| 19 | #1.17 NOT #1.18 | 2,352 |
| 20 | 'morbidity'/exp OR 'mortality'/exp OR morbidity:ab,ti OR mortality:ab,ti | 991,277 |
| 21 | 'survival'/exp OR 'prognosis'/exp OR 'follow up'/exp | 1,439,585 |
| 22 | prognos*:ab,ti OR predict*:ab,ti OR course*:ab,ti | 1,935,874 |
| 23 | 'death'/exp OR 'treatment outcome'/exp | 1,305,154 |
| 24 | ((disease OR disorder) NEAR/3 (incidence OR frequency)):ab,ti | 23,564 |
| 25 | death:ab,ti OR exitus:ab,ti | 553,722 |
| 26 | relaps*:ab,ti OR recrudescence:ab,ti OR deteriorat*:ab,ti OR exacerbat*:ab,ti OR worse*:ab,ti OR recover*:ab,ti OR recurren*:ab,ti | 1,399,746 |
| 27 | cure*:ab,ti OR curative*:ab,ti OR resolv*:ab,ti OR resolution*:ab,ti OR heal*:ab,ti OR improv*:ab,ti OR recuperat*:ab,ti OR survival:ab,ti | 4,446,912 |
| 28 | #1.20 OR #1.21 OR #1.22 OR #1.23 OR #1.24 OR #1.25 OR #1.26 OR #1.27 | 8,011,056 |
| 29 | #1.19 AND #1.28 | 1,480 |
| 30 | 'mental disease'/exp OR 'cardiovascular disease'/exp OR 'musculoskeletal disease'/exp OR 'digestive system disease'/exp AND 'thorax disease'/exp OR 'respiratory tract disease'/exp | 1,731,480 |
| 31 | ((heart OR cardiac OR cardiovascular OR vascular OR coronary OR thoracic OR thorax OR pulmonary OR lung OR bronchial OR muscoskeletal OR digestive OR gastrointestinal OR mental OR psycho*) NEAR/3 (disease* OR disorder*)):ab,ti | 603,119 |
| 32 | 'heart failure':ab,ti OR stroke:ab,ti OR cox:ab,ti | 417,737 |
| 33 | #1.30 OR #1.31 OR #1.32 | 2,520,128 |
| 34 | #1.29 AND #1.33 | 1,166 |
| 35 | #1.29 AND #1.33 AND ([embryo]/lim OR [fetus]/lim OR [newborn]/lim OR [infant]/lim OR [preschool]/lim OR [school]/lim OR [child]/lim OR [adolescent]/lim) | 104 |
| 36 | #1.29 AND #1.33 AND ([embryo]/lim OR [fetus]/lim OR [newborn]/lim OR [infant]/lim OR [preschool]/lim OR [school]/lim OR [child]/lim OR [adolescent]/lim) AND ([adult]/lim OR [aged]/lim) | 77 |
| 37 | #1.35 NOT #1.36 | 27 |
| 38 | #1.34 NOT #1.37 | 1,139 |
| 39 | #1.34 NOT #1.37 AND [animals]/lim | 10 |
| 40 | #1.34 NOT #1.37 AND [animals]/lim AND [humans]/lim | 9 |
| 41 | #1.39 NOT #1.40 | 1 |
| 42 | #1.38 NOT #1.41 AND [1993-2014]/py | 1,095 |
