# Supplementary material for: Treatment Efficacy for Non-Cardiovascular Chest Pain: A Systematic Review and Meta-Analysis
Source: PLoS One. 2014 Aug 11;9(8):e104722. doi: 10.1371/journal.pone.0104722 (PMC4128723; doi:10.1371/journal.pone.0104722)
Supplement: Table S2 — Summary of the SIGN quality assessment. (DOCX) [file pone.0104722.s003.docx]

Table S2: Summary of the SIGN quality assessment [[68](#_ENREF_68)]

| **Author** | **Year** | **1.1** | **1.2** | **1.3** | **1.4** | **1.5** | **1.6** | **1.7** | **1.8** | **1.9** | **1.10.** | **2.1** |
| --- | --- | --- | --- | --- | --- | --- | --- | --- | --- | --- | --- | --- |
| **GI-Symptoms** |  |  |  |  |  |  |  |  |  |  |  |  |
| Achem | 1997 | yes | yes | yes | yes | can't say | can't say | yes | 5.6% | no | does not apply | (+) |
| Bautista | 2004 | yes | yes | yes | yes | yes | yes | yes | no | yes | does not apply | (++) |
| Cossentino | 2012 | yes | yes | yes | yes | yes | yes | yes | Placebo: 10.0%, Control: 30.0% | yes | does not apply | (+) |
| Dickman | 2005 | yes | yes | yes | yes | yes | yes | yes | no | yes | does not apply | (++) |
| Dore | 2007 | yes | yes | yes | can't say | can't say | can't say | yes | 10.9% | no | does not apply | (+) |
| Fass | 1998 | yes | yes | yes | yes | can't say | yes | yes | no | yes | does not apply | (+) |
| Jones | 2006 | yes | yes | can't say | yes | can't say | can't say | yes | 7.1% | yes | does not apply | (+) |
| Lind | 1997 | yes | yes | yes | yes | yes | yes | yes | 4.7% | yes | can't say | (++) |
| Pandak | 2002 | yes | yes | can't say | yes | can't say | yes | yes | 11.9% | yes | does not apply | (+) |
| Xia | 2003 | yes | yes | no | yes | yes | can't say | yes | 2.9% | can't say | does not apply | (+) |
| **musculoskeletal** |  |  |  |  |  |  |  |  |  |  |  |  |
| Lethola | 2010 | yes | yes | no | yes | yes | can't say | yes | 4.4% | no | can't say | (+) |
| Stochkendahl | 2012 | yes | yes | yes | can't say | yes | yes | yes | Chiropractic: 15.2%, Self-management: 28.6% | yes | can't say | (+) |
| **psychotropic drugs** |  |  |  |  |  |  |  |  |  |  |  |  |
| Cannon | 1994 | yes | yes | can't say | yes | can't say | yes | yes | no | yes | does not apply | (+) |
| Cox | 1998 | yes | yes | can't say | yes | can't say | yes | yes | 16.7% | can't say | does not apply | (+) |
| Doraiswamy | 2006 | yes | yes | can't say | yes | yes | yes | yes | Paroxetine: 18.5%. Placebo: 8.7% | can't say | does not apply | (+) |
| Keefe | 2011 | yes | yes | yes | yes | can't say | yes | yes | 23.3% | yes | can't say | (+) |
| Rao | 2007 | yes | yes | yes | yes | can't say | can't say | yes | 20.8% | can't say | does not apply | (+) |
| Varia | 2000 | yes | yes | yes | yes | yes | yes | yes | 16.7% | yes | does not apply | (+) |
| Wulsin | 2002 | yes | yes | no | no | can't say | can't say | yes | 14.1% | no | does not apply | (+) |
| **psychological interventions** |  |  |  |  |  |  |  |  |  |  |  |  |
| Arnold | 2009 | yes | yes | yes | no | yes | yes | yes | 29.40% | can't say | does not apply | (+) |
| Esler | 2003 | yes | yes | yes | no | yes | can't say | yes | 39.0% | yes | does not apply | (+) |
| Gasiorowska | 2008 | yes | yes | can't say | can't say | yes | can't say | yes | 26.4% | can't say | no | (+) |
| Hess | 2012 | yes | yes | yes | yes | yes | can't say | yes | 1.9% | yes | does not apply | (++) |
| Jonsbu | 2011 | yes | yes | yes | no | yes | can't say | yes | Intervention: 4.8%, Control: 5.3% | can't say | does not apply | (+) |
| Lahmann | 2008 | yes | yes | can't say | can't say | yes | can't say | yes | no | yes | does not apply | (+) |
| Mayou | 1997 | yes | yes | yes | no | yes | can't say | yes | CBT: 25.0%, Control: 41.2% | yes | does not apply | (+) |
| Mayou | 2002 | yes | yes | yes | yes | yes | yes | yes | 7.50% | yes | does not apply | (++) |
| Potts | 1999 | yes | yes | can't say | no | can't say | can't say | yes | 6.7% | yes | does not apply | (+) |
| Sanders | 1997 | yes | yes | can't say | yes | yes | can't say | yes | Treatment: 21.2%. Control: 37.5% | yes | does not apply | (+) |
| van Peski | 1999 | yes | can't say | no | no | can't say | can't say | yes | no | no | does not apply | (+) |

CBT: intervention based on cognitive behavioral principals; 1.1, appropriate and clearly focused question; 1.2, assignment is randomised; 1.3, adequate concealment; 1.4, blinding; 1.5; treatment and control groups are similar at baseline; 1.6, The treatment under investigation is the only difference; 1.7, all relevant outcomes are measured; 1.9, intention to treat analysis; 1.10, various sites are comparable; 2.1, risk of bias: (++), high quality: most of the criteria have been fulfilled. If not fulfilled, the conclusions of the study are very unlikely to alter; (+), moderate quality: some criteria fulfilled. Criteria not adequately described are unlikely to alter the conclusions; (-), low quality: few or no criteria fulfilled. The conclusions are likely to alter
