# Supplementary material for: Treatment Efficacy for Non-Cardiovascular Chest Pain: A Systematic Review and Meta-Analysis
Source: PLoS One. 2014 Aug 11;9(8):e104722. doi: 10.1371/journal.pone.0104722 (PMC4128723; doi:10.1371/journal.pone.0104722)
Supplement: Table S3 — Risk for Outcome Reporting Bias in Trials (ORBIT) in studies excluded from Meta-analyses. (DOCX) [file pone.0104722.s004.docx]

Table S3: Risk for Outcome Reporting Bias in Trials (ORBIT) in studies excluded from Meta-analyses [[20](#_ENREF_20)]

| **Author** | **Year** | **Treatment** | **ORBIT classification** | **Risk of ORB** | **reason** |
| --- | --- | --- | --- | --- | --- |
| Arnold | 2009 | CBT | Outcome was measured but not necessarily analyzed (not because of non-significant results) | low | not included because of missing baseline values |
| Sanders | 1997 | CBT | Outcome was measured but not necessarily analyzed (not because of non-significant results) | low | No pain intensity or frequency analyzed |
| Mayou | 2002 | CBT | Outcome was measured but not necessarily analyzed (not because of non-significant results) | low | NCCP small subgroup, no analysis for the subgroup and no analysis for the treatment group |
| Cox | 1998 | Imipramine | Outcome was measured but not necessarily analyzed (not because of non-significant results) | low | Missing baseline values |
| Dore | 2007 | PPI Therapy | Outcome was measured but not necessarily analyzed (not because of non-significant results) | low | NCCP small subgroup, no analysis for the subgroup and no analysis for the treatment group |
| Rao | 2007 | Theophyllin | Trial report states that outcome was analyzed but insufficient data were presented to be included in the meta-analysis | low | Insufficient results for the meta-analysis. Significantly less NCCP treatment compared to placebo |
| Lahmann | 2008 | Functional relaxation | Outcome was not measured | no | NCCP not measured |
| Hess | 2012 | Decision aid | Outcome was not measured | no | primary outcome knowledge of patients |
| Wulsin | 2002 | Pharmacologic Therapy | Outcome was not measured | no | NCCP not measured |
