# Supplementary material for: Treatment Efficacy for Non-Cardiovascular Chest Pain: A Systematic Review and Meta-Analysis
Source: PLoS One. 2014 Aug 11;9(8):e104722. doi: 10.1371/journal.pone.0104722 (PMC4128723; doi:10.1371/journal.pone.0104722)
Supplement: Table S4 — Detailed summary of results. (DOCX) [file pone.0104722.s005.docx]

Table S4: Detailed summary of results

| **Author** | **Year** | **n final total** | **n final group** | **Treatment** | **Treat-ment duration (weeks)** | **Outcome** | **Scale** | **Mean / median / n** | **Value (n / decrease)** | **SD** |
| --- | --- | --- | --- | --- | --- | --- | --- | --- | --- | --- |
| **GI-Symptoms** | | | | | | | | | | |
| Achem | 1997 | 34 | 16 | omeprazole 20 mg twice daily | 8 | ≥50% reduction of severity | categorical scale (0-10) | n | 7 |  |
| Achem | 1997 | 34 | 16 | placebo | 8 | ≥50% reduction of severity | categorical scale (0-10) | n | 3 |  |
| Bautista | 2004 | 40 | 18 | GERD-positive: lansoprazole (60 mg AM and 30 mg PM) | 1 | ≥50% symptom improvement | symptom intensity score = daily severity x daily frequency | n | 14 |  |
| Bautista | 2004 | 40 | 18 | GERD-positive: placebo | 1 | ≥50% symptom improvement | symptom intensity score = daily severity x daily frequency | n | 4 |  |
| Bautista | 2004 | 40 | 22 | GERD-negative lansoprazole (60 mg AM and 30 mg PM) | 1 | ≥50% symptom improvement | symptom intensity score = daily severity x daily frequency | n | 2 |  |
| Bautista | 2004 | 40 | 22 | GERD-negative: placebo | 1 | ≥50% symptom improvement | symptom intensity score = daily severity x daily frequency | n | 8 |  |
| Cossentino | 2012 | 34 | 23 | baclofen 10 mg | 2 | chest pain score | Likert numerical scale | median | 0.5 | n.r. |
| Cossentino | 2012 | 34 | 20 | placebo | 2 | chest pain score | Likert numerical scale | median | 0.5 | n.r. |
| Dickman | 2005 | 35 | 16 | GERD-positive: rabeprazole (20 mg am before breakfast and 20 mg pm before dinner) | 1 | ≥50% symptom improvement | symptom intensity score = daily severity x daily frequency | n | 12 |  |
| Dickman | 2005 | 35 | 16 | GERD-positive placebo | 1 | ≥50% symptom improvement | symptom intensity score = daily severity x daily frequency | n | 3 |  |
| Dickman | 2005 | 35 | 19 | GERD-negative: rabeprazole (20 mg am before breakfast and 20 mg pm before dinner) | 1 | ≥50% symptom improvement | symptom intensity score = daily severity x daily frequency | n | 2 |  |
| Dickman | 2005 | 35 | 19 | GERD-negative: placebo | 1 | ≥50% symptom improvement | symptom intensity score = daily severity x daily frequency | n | 4 |  |
| Dore | 2007 | 237 | 40 | Rabeprazole 20 mg vs pantoprazole 20 mg vs esomeprazole 20 mg vs lansoprazole 20 mg | 12 | Number of patients with chest pain | Presence of pain | n | n.r. |  |
| Fass | 1998 | 37 | 23 | GERD-positive: omeprazole (40 mg AM and 20 mg PM) | 1 | ≥50% symptom improvement | symptom score = daily severity x daily frequency | n | 18 |  |
| Fass | 1998 | 37 | 23 | GERD-positive: placebo | 1 | ≥50% symptom improvement | symptom score = daily severity x daily frequency | n | 6 |  |
| Fass | 1998 | 37 | 14 | GERD-negative: omeprazole (40 mg AM and 20 mg PM) | 1 | ≥50% symptom improvement | symptom score = daily severity x daily frequency | n | 2 |  |
| Fass | 1998 | 37 | 14 | GERD-negative: placebo | 1 | ≥50% symptom improvement | symptom score = daily severity x daily frequency | n | 1 |  |
| Jones | 2006 | 28 | 15 | hypnotherapy | 17 | improvement yes in chest pain | improvement = completely or moderately better / no improvement = slightly better, no change, slightly worse, moderately worse, much worse | n | 12 |  |
| Jones | 2006 | 28 | 13 | supportive therapy plus placebo | 17 | improvement yes in chest pain | improvement = completely or moderately better / no improvement = slightly better, no change, slightly worse, moderately worse, much worse | n | 3 |  |
| Jones | 2006 | 28 | 15 | hypnotherapy | 17 | change in pain scores | linear analogue scale 0-100 | mean | 30.57 | 15.27 |
| Jones | 2006 | 28 | 13 | supportive therapy plus placebo | 17 | change in pain scores | linear analogue scale 0-101 | mean | 13.31 | 16.28 |
| Jones | 2006 | 28 | 15 | hypnotherapy | 17 | change in pain frequency scores | pain episodes per month | median | 12 | n.r. |
| Jones | 2006 | 28 | 13 | supportive therapy plus placebo | 17 | change in pain frequency scores | pain episodes per month | median | 20 | n.r. |
| Lind | 1997 | 485 | 205 | omeprazole 20 mg once daily | 4 | ≥50% heartburn improvement | n.r. | n | 125 |  |
| Lind | 1997 | 485 | 199 | omeprazole 10 mg once daily | 4 | ≥50% heartburn improvement | n.r. | n | 98 |  |
| Lind | 1997 | 485 | 105 | placebo | 4 | ≥50% heartburn improvement | n.r. | n | 25 |  |
| Pandak | 2002 | 37 | 22 | Sequence 1: 40 mg of omeprazole orally twice daily | 2 | improvement in treatment score | VAS | mean | 2.91 | 2.39 |
| Pandak | 2002 | 37 | 22 | Sequence 1: placebo for 2 weeks | 2 | improvement in treatment score | VAS | mean | 0.73 | 2.51 |
| Pandak | 2002 | 37 | 20 | Sequence 2: 40 mg of omeprazole orally twice daily | 2 | improvement in treatment score | VAS | mean | 4.67 | 2.34 |
| Pandak | 2002 | 37 | 20 | Sequence 2: placebo for 2 weeks | 2 | improvement in treatment score | VAS | mean | 0.93 | 2.57 |
| Pandak | 2002 | 37 | 20 | GERD-positive: omeprazole (40 mg orally twice daily) | 2 | ≥50% response | n.r. | n | 18 |  |
| Pandak | 2002 | 37 | 20 | GERD-positive: placebo | 2 | ≥50% response | n.r. | n | 1 |  |
| Pandak | 2002 | 37 | 18 | GERD-negative: omeprazole (40 mg orally twice daily) | 2 | ≥50% response | n.r. | n | 6 |  |
| Pandak | 2002 | 37 | 18 | GERD-negative: placebo | 2 | ≥50% response | n.r. | n | 3 |  |
| Xia | 2003 | 68 | 36 | lansoprazole 30 mg daily | 4 | ≥50% chest pain symptom improvement | symptom score = severity x frequency | n | 34 |  |
| Xia | 2003 | 68 | 32 | placebo daily | 4 | ≥50% chest pain symptom improvement | symptom score = severity x frequency | n | 20 |  |
| Xia | 2003 | 68 | 36 | lansoprazole 30 mg daily | 4 | symptom score | symptom score = severity x frequency (1-20) | mean | 2.05 | 0.33 |
| Xia | 2003 | 68 | 32 | placebo daily | 4 | symptom score | symptom score = severity x frequency (1-20) | mean | 2 | 0.28 |
| **musculoskeletal** | | | | | | | | | | |
| Lehtola | 2010 | 109 | 37 | Facettraction manipulation: manipulate as many segments between the areas T3–T8 as considered necessary. The treatment time lasted 10–15 min. This technique is widely used among OMT, physiotherapists and osteopaths | 3 | decrease in VAS Score | VAS 0-10 | mean | 2 | 2.14 |
| Lehtola | 2010 | 109 | 35 | Acupuncture: points GB21, GV14, GV9–12, BL13–18 l.a., BL60 l.a. Points GV9–12 were used so that needling occurred at a segment above, and to a segment below, the symptomatic segment. The duration of treatment was 30 min | 3 | decrease in VAS Score | VAS 0-10 | mean | 2.5 | 1.85 |
| Lehtola | 2010 | 109 | 37 | Placebo: interference electrotherapy with suction cups in place but without electricity. The effect of intermittent sucking varied between 0–0.2 bar. Each treatment lasted for 20 min. | 3 | decrease in VAS Score | VAS 0-10 | mean | 2.9 | 1.68 |
| Stochkendahl | 2012 | 90 | 38 | Chiropractic treatment: 1 of 8 experienced chose an individual treatment strategy, treatment had to include high-velocity, low-amplitude manipulation directed toward the thoracic and/or cervical spine, max. 10 sessions, 1-3 times per week for 4 weeks | 4 | % with pain, Complaint: Chest pain now | 11-point numeric rating scale (0-10) | n | 14 |  |
| Stochkendahl | 2012 | 90 | 35 | Self-management: told that chest pain generally had a benign, self-limiting course and, based on the clinical evaluation, individual instructions regarding posture and 2 to 3 home exercises aimed at increasing spinal movement or muscle stretch | 4 | % with pain, Complaint: Chest pain now | 11-point numeric rating scale (0-10) | n | 12 |  |
| Stochkendahl | 2012 | 90 | 48 | Chiropractic treatment: 1 of 8 experienced chose an individual treatment strategy, treatment had to include high-velocity, low-amplitude manipulation directed toward the thoracic and/or cervical spine, max. 10 sessions, 1-3 times per week for 4 weeks | 4 | decrease in chest pain average | 11-point numeric rating scale (0-10) | mean | 2.88 | 2.13 |
| Stochkendahl | 2012 | 90 | 37 | Self-management: told that chest pain generally had a benign, self-limiting course and, based on the clinical evaluation, individual instructions regarding posture and 2 to 3 home exercises aimed at increasing spinal movement or muscle stretch | 4 | decrease in chest pain average | 11-point numeric rating scale (0-10) | mean | 3.08 | 2.27 |
| Stochkendahl | 2012 | 90 | 55 | Chiropractic treatment: 1 of 8 experienced chose an individual treatment strategy, treatment had to include high-velocity, low-amplitude manipulation directed toward the thoracic and/or cervical spine, max. 10 sessions, 1-3 times per week for 4 weeks | 4 | decrease in chest pain average | 11-point numeric rating scale (0-10) | mean | 2.12 | 2.03 |
| Stochkendahl | 2012 | 90 | 44 | Self-management: told that chest pain generally had a benign, self-limiting course and, based on the clinical evaluation, individual instructions regarding posture and 2 to 3 home exercises aimed at increasing spinal movement or muscle stretch | 4 | decrease in chest pain average | 11-point numeric rating scale (0-10) | mean | 2.21 | 1.81 |
| **psychotropic drugs** | | | | | | | | | | |
| Cannon | 1994 | 60 | 20 | Placebo with chest pain during right ventricular stimulation at base line and during treatment phase | 3 | CGIS | Clinical Global Improvement Scale (CGIS) (0-7) Chest pain | mean | 1.2 | 1.63 |
| Cannon | 1994 | 60 | 20 | Clonidine 0.1 mg with chest pain during right ventricular stimulation at base line and during treatment phase | 3 | CGIS | Clinical Global Improvement Scale (CGIS) (0-7) Chest pain | mean | 2 | 2.72 |
| Cannon | 1994 | 60 | 20 | Imipramine 50 mg with chest pain during right ventricular stimulation at base line and during treatment phase | 3 | CGIS | Clinical Global Improvement Scale (CGIS) (0-7) Chest pain | mean | 2.4 | 3.26 |
| Cannon | 1994 | 60 | 20 | Placebo with chest pain during right ventricular stimulation at base line and during treatment phase | 3 | pain | yes/no | n | 14 |  |
| Cannon | 1994 | 60 | 20 | Clonidine 0.1 mg with chest pain during right ventricular stimulation at base line and during treatment phase | 3 | pain | yes/no | n | 12 |  |
| Cannon | 1994 | 60 | 20 | Imipramine 50 mg with chest pain during right ventricular stimulation at base line and during treatment phase | 3 | pain | yes/no | n | 6 |  |
| Cox | 1998 | 15 | 15 | Imipramine 50 mg daily vs. Placebo | 5 | Pain severity at follow-up | Pain scale 0-10 | n.r. | n.r. | n.r. |
| Dorais-wamy | 2006 | n.r. | n.r. | Paroxetine: 10 mg for 1 week, then increased to 20 mg daily, and further titrated based on clinical response by the study physician to a maximum daily dose of 50 mg | 9 | VAS (0-10) | severity chest pain | mean | 12.5 | 11.73 |
| Dorais-wamy | 2006 | n.r. | n.r. | Placebo | 9 | VAS (0-10) | severity chest pain | mean | 14 | 20.62 |
| Keefe | 2011 | 115 | 28 | placebo alone | 34 | pain intensity | visual analog scale (0-100) | mean | 7.94 | 12.40 |
| Keefe | 2011 | 115 | 30 | Sertraline alone (started at 50 mg per day and the dose was titrated to a maximum of 200 mg over the course of the initial 10 weeks of treatment. Dosage was adjusted by the study psychiatrist based on clinical response. After the initial 10 weeks of treatment, the dose level was stabilized for the remaining 24 weeks of the study) | 34 | pain intensity | visual analog scale (0-100) | mean | 7.26 | 9.33 |
| Keefe | 2011 | 115 | 29 | CST (CST was delivered in 5, 60-min individual sessions held bi-weekly for 10 weeks and 6, 30-min individual follow-up sessions held monthly for 6 months.) plus placebo | 34 | pain intensity | visual analog scale (0-100) | mean | 10.71 | 14.07 |
| Keefe | 2011 | 115 | 28 | CST (CST was delivered in 5, 60-min individual sessions held bi-weekly for 10 weeks and 6, 30-min individual follow- up sessions held monthly for 6 months.) plus sertraline (started at 50 mg per day and the dose was titrated to a maximum of 200 mg over the course of the initial 10 weeks of treatment. Dosage was adjusted by the study psychiatrist based on clinical response. After the initial 10 weeks of treatment, the dose level was stabilized for the remaining 24 weeks of the study.) | 34 | pain intensity | visual analog scale (0-100) | mean | 6.67 | 15.45 |
| Keefe | 2011 | 115 | 28 | placebo alone | 34 | pain unpleasantness | visual analog scale (0-100) | mean | 8.39 | 14.57 |
| Keefe | 2011 | 115 | 30 | Sertraline alone (started at 50 mg per day and the dose was titrated to a maximum of 200 mg over the course of the initial 10 weeks of treatment. Dosage was adjusted by the study psychiatrist based on clinical response. After the initial 10 weeks of treatment, the dose level was stabilized for the remaining 24 weeks of the study) | 34 | pain unpleasantness | visual analog scale (0-100) | mean | 7.61 | 9.56 |
| Keefe | 2011 | 115 | 29 | CST (CST was delivered in 5, 60-min individual sessions held bi-weekly for 10 weeks and 6, 30-min individual follow-up sessions held monthly for 6 months.) plus placebo | 34 | pain unpleasantness | visual analog scale (0-100) | mean | 12.1 | 15.20 |
| Keefe | 2011 | 115 | 28 | CST (CST was delivered in 5, 60-min individual sessions held bi-weekly for 10 weeks and 6, 30-min individual follow- up sessions held monthly for 6 months.) plus sertraline (started at 50 mg per day and the dose was titrated to a maximum of 200 mg over the course of the initial 10 weeks of treatment. Dosage was adjusted by the study psychiatrist based on clinical response. After the initial 10 weeks of treatment, the dose level was stabilized for the remaining 24 weeks of the study.) | 34 | pain unpleasantness | visual analog scale (0-100) | mean | 6.73 | 15.37 |
| Rao | 2007 | 19 | 19 | theophylline 200 mg daily | 4 | severity of chest pain | Likert numerical scale (0-3) | median | n.r. | n.r. |
| Rao | 2007 | 19 | 19 | placebo | 4 | severity of chest pain | Likert numerical scale (0-3) | median | n.r. | n.r. |
| Varia | 2000 | 30 | 15 | Sertraline. Doses started at 50 mg and were adjusted to a maximum of 200 mg | 8 | average pain | visual analogue scale (VAIS-R) (0-10) | mean | 2.47 | 1.94 |
| Varia | 2000 | 30 | 15 | placebo | 8 | average pain | visual analogue scale (VAIS-R) (0-10) | mean | 0.54 | 2.71 |
| Wulsin | 2002 | 50 | 25 | Usual care: reassurance that the patient has no cardiac disease causing the chest pain and discharge to care as needed through the patient’s primary care physician (PCP). | 4 | panic disorder severity scale | interview, 7 item (0-5) | mean | 1.52 | n.r. |
| Wulsin | 2002 | 50 | 25 | Intervention: patient education by the research assistant about panic disorder according to study protocol, initiation of treatment by the emergency physician with a 1-month supply of antipanic medication (paroxetine 20 mg/d) | 4 | panic disorder severity scale | interview, 7 item (0-5) | mean | 1.26 | n.r. |
| Wulsin | 2002 | 50 | 25 | Usual care: reassurance that the patient has no cardiac disease causing the chest pain and discharge to care as needed through the patient’s primary care physician (PCP). | 4 | CMID >=40% reduction in pdss | n.r. | n | 9.25 |  |
| Wulsin | 2002 | 50 | 25 | Intervention: patient education by the research assistant about panic disorder according to study protocol, initiation of treatment by the emergency physician with a 1-month supply of antipanic medication (paroxetine 20 mg/d) | 4 | CMID >=40% reduction in pdss | n.r. | n | 8.25 |  |
| **psychological interventions** | | | | | | | | | | |
| Arnold | 2009 | 494 | 246 | verbal advice augmented with an information sheet | 0 | Chest pain | Scale 0-10 | mean | n.r. | n.r. |
| Arnold | 2009 | 494 | 248 | verbal advice alone | 0 | Chest pain | Scale 0-10 | mean | n.r. | n.r. |
| Esler | 2003 | 36 | 17 | CBT, 60min, 1 psychologist | 0 | Chest pain severity | 0-8 scale | mean | 3.6 | 1.26 |
| Esler | 2003 | 36 | 19 | control | 0 | Chest pain severity | 0-8 scale | mean | 4.1 | 1.28 |
| Esler | 2003 | 36 | 17 | CBT, 60min, 1 psychologist | 0 | Chest pain frequency past month | n per month | mean | 10.4 | 11.75 |
| Esler | 2003 | 36 | 19 | control | 0 | Chest pain frequency past month | n per month | mean | 3.4 | 7.56 |
| Gasiorowska | 2008 | 39 | 21 | Johrei treatment, 20min | 6 | decrease in chest pain symptom intensity | symptom score = daily severity x daily frequency | mean | 13.28 | n.r. |
| Gasiorowska | 2008 | 39 | 18 | waiting list | 6 | decrease in chest pain symptom intensity | symptom score = daily severity x daily frequency | mean | 2.37 | n.r. |
| Hess | 2012 | 204 | 101 | decision aid | 0 | Cardiac stress testing performed | n.a. | n | 75 |  |
| Hess | 2012 | 204 | 103 | usual care | 0 | Cardiac stress testing performed | n.a. | n | 94 |  |
| Hess | 2012 | 204 | 101 | decision aid | 0 | Coronary revascularization | n.a. | n | 3 |  |
| Hess | 2012 | 204 | 103 | usual care | 0 | Coronary revascularization | n.a. | n | 2 |  |
| Hess | 2012 | 204 | 101 | decision aid | 0 | Management decision: Observation unit admission and cardiac stress testing | n.a. | n | 58 |  |
| Hess | 2012 | 204 | 103 | usual care | 0 | Management decision: Observation unit admission and cardiac stress testing | n.a. | n | 77 |  |
| Hess | 2012 | 204 | 101 | decision aid | 0 | Management decision: Follow-up with a cardiologist + Follow-up with a primary-care physician | n.a. | n | 39 |  |
| Hess | 2012 | 204 | 103 | usual care | 0 | Management decision: Follow-up with a cardiologist + Follow-up with a primary-care physician | n.a. | n | 9 |  |
| Hess | 2012 | 204 | 101 | decision aid | 0 | Repeat emergency department visit + rehospitalization | n.a. | n | 5 |  |
| Hess | 2012 | 204 | 103 | usual care | 0 | Repeat emergency department visit + rehospitalization | n.a. | n | 0 |  |
| Jonsbu | 2011 | 38 | 20 | Intervention: Three 60– 90-min CBT sessions at the Psychiatric Outpatient Clinic at Molde Hospital | n.r. | Frequency of symptoms of chest pain or palpitations | 1-4 (1 (“daily”), 2 (“weekly or more often”), 3 (“rare but sometimes”), and 4 (“no symptoms”)) | mean | 0.46 | 0.46 |
| Jonsbu | 2011 | 38 | 18 | Control: normal care from their general practitioner, free use of health care system when needed | n.r. | Frequency of symptoms of chest pain or palpitations | 1-4 (1 (“daily”), 2 (“weekly or more often”), 3 (“rare but sometimes”), and 4 (“no symptoms”)) | mean | 0.18 | 0.52 |
| Lahmann | 2008 | 22 | 11 | functional relaxation | 6 | Cardiovascular Complaints (Giessen) | 5-point Likert Scale | mean | 12.65 | 14.62 |
| Lahmann | 2008 | 22 | 11 | enhanced medical care | 6 | Cardiovascular Complaints (Giessen) | 5-point Likert Scale | mean | -0.5 | 13.73 |
| Mayou | 1997 | 25 | 15 | CBT, up to 12 sessions | n.r. | Symptoms Severity (last month) | n.r. | mean | 1.05 | 0.80 |
| Mayou | 1997 | 25 | 10 | Control | n.r. | Symptoms Severity (last month) | n.r. | mean | 0.23 | 0.93 |
| Mayou | 1997 | 25 | 15 | CBT, up to 12 sessions | n.r. | Symptoms Frequency (last month) | last month | mean | 0.70 | 1.04 |
| Mayou | 1997 | 25 | 10 | Control | n.r. | Symptoms Frequency (last month) | last month | mean | 0.82 | 1.26 |
| Mayou | 2002 | 64 | 31 | CBT | n.r. | Frequency of chest pain | bad = 3, good = 0 | mean | n.r. | n.r. |
| Mayou | 2002 | 64 | 33 | Control | n.r. | Frequency of chest pain | bad = 3, good = 0 | mean | n.r. | n.r. |
| Potts | 1999 | 56 | 32 | Immediate psychological treatment | 8 | Chest pain Episodes/week |  | median | 3 | n.r. |
| Potts | 1999 | 56 | 24 | waiting control | 8 | Chest pain Episodes/week |  | median | 0 | n.r. |
| Potts | 1999 | 56 | 32 | Immediate psychological treatment | 8 | Chest pain Duration (min) |  | median | 1.6 | n.r. |
| Potts | 1999 | 56 | 24 | waiting control | 8 | Chest pain Duration (min) |  | median | 0.5 | n.r. |
| Potts | 1999 | 56 | 32 | Immediate psychological treatment | 8 | Chest pain Severity | Scale 1-100 | median | 5.9 | n.r. |
| Potts | 1999 | 56 | 24 | waiting control | 8 | Chest pain Severity | Scale 1-100 | median | -0.8 | n.r. |
| Sanders | 1997 | 50 | 29 | Intervention: 1-hour individualized information | 0 | Number of patients with chest pain | daily or at least weekly | n | 5 |  |
| Sanders | 1997 | 50 | 21 | Control | 0 | Number of patients with chest pain | daily or at least weekly | n | 4 |  |
| Sanders | 1997 | 50 | 29 | Intervention: 1-hour individualized information | 0 | Number of patients with moderate to severe chest pain | Moderately to very severe | n | 11 |  |
| Sanders | 1997 | 50 | 21 | Control | 0 | Number of patients with moderate to severe chest pain | Moderately to very severe | n | 5 |  |
| van Peski | 1999 | 65 | 32 | CBT, 4-12 sessions weekly | 26 | Intensity | Scale 0-10 | mean | 2.00 | 1.33 |
| van Peski | 1999 | 65 | 33 | Control | 26 | Intensity | Scale 0-10 | mean | 0.40 | 1.30 |
| van Peski | 1999 | 65 | 32 | CBT, 4-12 sessions weekly | 26 | Frequency | weekly | mean | 4.6 | 1.70 |
| van Peski | 1999 | 65 | 33 | Control | 26 | Frequency | weekly | mean | 0.1 | 4.38 |
| **Anxiety and Depression** | | | | | | | | | | |
| Arnold | 2009 | 237 | 108 | verbal advice augmented with an information sheet | 0 | HADS (mild - severe, 8-21) anxiety | Scale 0-3, 7 items, 0-21 | n | n.r. |  |
| Arnold | 2009 | 237 | 134 | verbal advice alone | 0 | HADS (mild - severe, 8-21) anxiety | Scale 0-3, 7 items, 0-21 | n | n.r. |  |
| Arnold | 2009 | 237 | 47 | verbal advice augmented with an information sheet | 0 | HADS (mild - severe, 8-21) depression | Scale 0-3, 7 items, 0-21 | n | n.r. |  |
| Arnold | 2009 | 237 | 65 | verbal advice alone | 0 | HADS (mild - severe, 8-21) depression | Scale 0-3, 7 items, 0-21 | n | n.r. |  |
| Cannon | 1994 | 60 | 20 | Placebo with chest pain during right ventricular stimulation at base line and during treatment phase | 3 | (Spielberger) State-Trait Anxiety Scale | Scale 1-4, 40 items (state anxiety 20 items, trait anxiety 20 items), 20-80 | mean | 4 | 5.43637699 |
| Cannon | 1994 | 60 | 20 | Clonidine 0.1 mg with chest pain during right ventricular stimulation at base line and during treatment phase | 3 | (Spielberger) State-Trait Anxiety Scale | Scale 1-4, 40 items (state anxiety 20 items, trait anxiety 20 items), 20-81 | mean | 3 | 4.07728275 |
| Cannon | 1994 | 60 | 20 | Imipramine 50 mg with chest pain during right ventricular stimulation at base line and during treatment phase | 3 | (Spielberger) State-Trait Anxiety Scale | Scale 1-4, 40 items (state anxiety 20 items, trait anxiety 20 items), 20-82 | mean | 5 | 6.79547124 |
| Dorais-wamy | 2006 | n.r. | n.r. | Paroxetine: 10 mg for 1 week, then increased to 20 mg daily, and further titrated based on clinical response by the study physician to a maximum daily dose of 50 mg | 9 | BDI | Scale 0-3, 21 items, 0-63 | n.r. | n.r. |  |
| Dorais-wamy | 2006 | n.r. | n.r. | Placebo | 9 | BDI | Scale 0-3, 21 items, 0-63 | n.r. | n.r. |  |
| Esler | 2003 | 36 | 17 | CBT, 60min, 1 psychologist | 0 | Anxiety Sensitivity Index | Scale 0-4, 16 items, 0-64 | mean | 6.2 | 8.25324179 |
| Esler | 2003 | 36 | 19 | control | 0 | Anxiety Sensitivity Index | Scale 0-4, 16 items, 0-64 | mean | -0.6 | 7.3545904 |
| Esler | 2003 | 36 | 17 | CBT, 60min, 1 psychologist | 0 | BSI Anxiety | Scale 0-4, 6 items, ?? | mean | 4.7 | 7.5230313 |
| Esler | 2003 | 36 | 19 | control | 0 | BSI Anxiety | Scale 0-4, 6 items, ?? | mean | -0.2 | 7.78151656 |
| Jones | 2006 | 28 | 15 | hypnotherapy | 17 | change in anxiety scores (HADS) | Scale 0-3, 7 items, 0-21 | median | 2 | 1.39140217 |
| Jones | 2006 | 28 | 13 | supportive therapy plus placebo | 17 | change in anxiety scores (HADS) | Scale 0-3, 7 items, 0-21 | median | -1.5 | 2.50599282 |
| Jones | 2006 | 28 | 15 | hypnotherapy | 17 | change in depression scores (HADS) | Scale 0-3, 7 items, 0-21 | median | -0.5 | 1.21655251 |
| Jones | 2006 | 28 | 13 | supportive therapy plus placebo | 17 | change in depression scores (HADS) | Scale 0-3, 7 items, 0-21 | median | -0.5 | 1.59248234 |
| Jonsbu | 2011 | 38 | 20 | Intervention: Three 60– 90-min CBT sessions at the Psychiatric Outpatient Clinic at Molde Hospital | n.r. | BDI | Scale 0-3, 21 items, 0-63 | mean | 3.4 | 4.10682359 |
| Jonsbu | 2011 | 38 | 18 | Control: normal care from their general practitioner, free use of health care system when needed | n.r. | BDI | Scale 0-3, 21 items, 0-63 | mean | -1.6 | 6.36380389 |
| Keefe | 2011 | 115 | 28 | placebo alone | 34 | State-Trait Anxiety Inventory | Scale 1-4, 40 items (state anxiety 20 items, trait anxiety 20 items), 20-80 | mean | 0.85 | 11.4999242 |
| Keefe | 2011 | 115 | 30 | Sertraline alone (started at 50 mg per day and the dose was titrated to a maximum of 200 mg over the course of the initial 10 weeks of treatment. Dosage was adjusted by the study psychiatrist based on clinical response. After the initial 10 weeks of treatment, the dose level was stabilized for the remaining 24 weeks of the study) | 34 | State-Trait Anxiety Inventory | Scale 1-4, 40 items (state anxiety 20 items, trait anxiety 20 items), 20-80 | mean | 3.89 | 10.2804123 |
| Keefe | 2011 | 115 | 29 | CST (CST was delivered in 5, 60-min individual sessions held bi-weekly for 10 weeks and 6, 30-min individual follow-up sessions held monthly for 6 months.) plus placebo | 34 | State-Trait Anxiety Inventory | Scale 1-4, 40 items (state anxiety 20 items, trait anxiety 20 items), 20-80 | mean | 7.65 | 12.2124127 |
| Keefe | 2011 | 115 | 28 | CST (CST was delivered in 5, 60-min individual sessions held bi-weekly for 10 weeks and 6, 30-min individual follow- up sessions held monthly for 6 months.) plus sertraline (started at 50 mg per day and the dose was titrated to a maximum of 200 mg over the course of the initial 10 weeks of treatment. Dosage was adjusted by the study psychiatrist based on clinical response. After the initial 10 weeks of treatment, the dose level was stabilized for the remaining 24 weeks of the study.) | 34 | State-Trait Anxiety Inventory | Scale 1-4, 40 items (state anxiety 20 items, trait anxiety 20 items), 20-80 | mean | 12.07 | 7.49589541 |
| Keefe | 2011 | 115 | 28 | placebo alone | 34 | BDI | Scale 0-3, 21 items, 0-63 | mean | 4.48 | 5.17935903 |
| Keefe | 2011 | 115 | 30 | Sertraline alone (started at 50 mg per day and the dose was titrated to a maximum of 200 mg over the course of the initial 10 weeks of treatment. Dosage was adjusted by the study psychiatrist based on clinical response. After the initial 10 weeks of treatment, the dose level was stabilized for the remaining 24 weeks of the study) | 34 | BDI | Scale 0-3, 21 items, 0-63 | mean | 2.05 | 5.08575461 |
| Keefe | 2011 | 115 | 29 | CST (CST was delivered in 5, 60-min individual sessions held bi-weekly for 10 weeks and 6, 30-min individual follow-up sessions held monthly for 6 months.) plus placebo | 34 | BDI | Scale 0-3, 21 items, 0-63 | mean | 3.16 | 5.54278811 |
| Keefe | 2011 | 115 | 28 | CST (CST was delivered in 5, 60-min individual sessions held bi-weekly for 10 weeks and 6, 30-min individual follow- up sessions held monthly for 6 months.) plus sertraline (started at 50 mg per day and the dose was titrated to a maximum of 200 mg over the course of the initial 10 weeks of treatment. Dosage was adjusted by the study psychiatrist based on clinical response. After the initial 10 weeks of treatment, the dose level was stabilized for the remaining 24 weeks of the study.) | 34 | BDI | Scale 0-3, 21 items, 0-63 | mean | 4.88 | 5.66169056 |
| Lahmann | 2008 | 22 | 11 | functional relaxation | 6 | SCL-90 anxiety | Scale 0-4, ?? Items, ?? | mean | 7.3 | 4.02243707 |
| Lahmann | 2008 | 22 | 11 | enhanced medical care | 6 | SCL-90 anxiety | Scale 0-4, ?? Items, ?? | mean | -0.2 | 6.060033 |
| Lahmann | 2008 | 22 | 11 | functional relaxation | 6 | SCL-90 depression | Scale 0-4, ?? Items, ?? | mean | 3.2 | 4.0654643 |
| Lahmann | 2008 | 22 | 11 | enhanced medical care | 6 | SCL-90 depression | Scale 0-4, ?? Items, ?? | mean | -0.1 | 4.92686513 |
| Mayou | 2002 | 72 | 35 | CBT | n.r. | BDI | Scale 0-3, 21 items, 0-63 | median (IQR) | 2 | 3.27939905 |
| Mayou | 2002 | 72 | 37 | Control | n.r. | BDI | Scale 0-3, 21 items, 0-63 | median (IQR) | 3.25 | 7.51222141 |
| Mayou | 2002 | 72 | 35 | CBT | n.r. | State-Trait Anxiety Inventory | Scale 1-4, 40 items (state anxiety 20 items, trait anxiety 20 items), 20-80 | median (IQR) | 3.5 | 8.22855945 |
| Mayou | 2002 | 72 | 37 | Control | n.r. | State-Trait Anxiety Inventory | Scale 1-4, 40 items (state anxiety 20 items, trait anxiety 20 items), 20-80 | median (IQR) | 0.75 | 9.12194478 |
| Sanders | 1997 | 41 | 26 | Intervention: 1-hour individualized information | 0 | BDI | Scale 0-3, 21 items, 0-63 | mean | 1.16 | n.r. |
| Sanders | 1997 | 41 | 15 | Control | 0 | BDI | Scale 0-3, 21 items, 0-63 | mean | 0.93 | n.r. |
| Sanders | 1997 | 41 | 26 | Intervention: 1-hour individualized information | 0 | STAI-T | Scale 1-4, 40 items (state anxiety 20 items, trait anxiety 20 items), 20-80 | mean | -0.22 | n.r. |
| Sanders | 1997 | 41 | 15 | Control | 0 | STAI-T | Scale 1-4, 40 items (state anxiety 20 items, trait anxiety 20 items), 20-80 | mean | 4.4 | n.r. |
| Sanders | 1997 | 41 | 26 | Intervention: 1-hour individualized information | 0 | SCL-90 anxiety | Scale 0-4 | mean | 0.19 | n.r. |
| Sanders | 1997 | 41 | 15 | Control | 0 | SCL-90 anxiety | Scale 0-4 | mean | -0.03 | n.r. |
| Sanders | 1997 | 41 | 26 | Intervention: 1-hour individualized information | 0 | SCL-90 depression | Scale 0-4 | mean | 0.05 | n.r. |
| Sanders | 1997 | 41 | 15 | Control | 0 | SCL-90 depression | Scale 0-4 | mean | 0.28 | n.r. |
| van Peski | 1999 | 65 | 32 | CBT, 4-12 sessions weekly | 26 | HADS (Anxiety) | Scale 0-3, 7 items, 0-21 | mean | 3.4 | 2.74590604 |
| van Peski | 1999 | 65 | 33 | Control | 26 | HADS (Anxiety) | Scale 0-3, 7 items, 0-21 | mean | 0.7 | 2.5 |
| van Peski | 1999 | 65 | 32 | CBT, 4-12 sessions weekly | 26 | HADS (Depression) | Scale 0-3, 7 items, 0-21 | mean | 1.4 | 3.15784737 |
| van Peski | 1999 | 65 | 33 | Control | 26 | HADS (Depression) | Scale 0-3, 7 items, 0-21 | mean | -0.8 | 2.57720779 |
| Varia | 2000 | 30 | 15 | Sertraline. Doses started at 50 mg and were adjusted to a maximum of 200 mg | 8 | BDI | Scale 0-3, 21 items, 0-63 | mean | 0.54 | n.r. |
| Varia | 2000 | 30 | 15 | placebo | 8 | BDI | Scale 0-3, 21 items, 0-64 | mean | 2.07 | n.r. |
| Wulsin | 2002 | 50 | 25 | Intervention: patient education by the research assistant about panic disorder according to study protocol, initiation of treatment by the emergency physician with a 1-month supply of antipanic medication (paroxetine 20 mg/d) | 4 | panic disorder severity scale | interview, Scale 0-4, 7 items, 0-7 | mean | 0.75 | n.r. |
| Wulsin | 2002 | 50 | 25 | Usual care: reassurance that the patient has no cardiac disease causing the chest pain and discharge to care as needed through the patient’s primary care physician (PCP). | 4 | panic disorder severity scale | interview, Scale 0-4, 7 items, 0-7 | mean | 0.28 | n.r. |
